# Evidence of Recent Intricate Adaptation in Human Populations

**DOI:** 10.1371/journal.pone.0165870

**Published:** 2016-12-19

**Authors:** Leeyoung Park

**Affiliations:** Natural Science Research Institute, Yonsei University, Seoul, Korea; German Cancer Research Center (DKFZ), GERMANY

## Abstract

Recent human adaptations have shaped population differentiation in genomic regions containing putative functional variants, mostly located in predicted regulatory elements. However, their actual functionalities and the underlying mechanism of recent adaptation remain poorly understood. In the current study, regions of genes and repeats were investigated for functionality depending on the degree of population differentiation, F_ST_ or ΔDAF (a difference in derived allele frequency). The high F_ST_ in the 5´ or 3´ untranslated regions (UTRs), in particular, confirmed that population differences arose mainly from differences in regulation. Expression quantitative trait loci (eQTL) analyses using lymphoblastoid cell lines indicated that the majority of the highly population-specific regions represented cis- and/or trans-eQTL. However, groups having the highest ΔDAFs did not necessarily have higher proportions of eQTL variants; in these groups, the patterns were complex, indicating recent intricate adaptations. The results indicated that East Asian (EAS) and European populations (EUR) experienced mutual selection pressures. The mean derived allele frequency of the high ΔDAF groups suggested that EAS and EUR underwent strong adaptation; however, the African population in Africa (AFR) experienced slight, yet broad, adaptation. The DAF distributions of variants in the gene regions showed clear selective pressure in each population, which implies the existence of more recent regulatory adaptations in cells other than lymphoblastoid cell lines. In-depth analysis of population-differentiated regions indicated that the coding gene, *RNF135*, represented a trans-regulation hotspot via cis-regulation by the population-specific variants in the region of selective sweep. Together, the results provide strong evidence of actual intricate adaptation of human populations via regulatory manipulation.

## Introduction

Recent large-scale human genome studies have revealed that all human beings share an almost identical genome [1–4]; however, minor differences among human populations exist as a result of genetic drift and adaptation. Using the genotyping data from the HapMap consortium [5], genome-wide F_ST_ estimates and a composite method involving F_ST_ have been used to identify genes under selection pressure [6–8]. It was found that genic regions harbored higher proportions of highly differentiated variants than non-genic regions, indicating selection pressure [6, 9]. Recent studies indicated that classic selective sweeps were rare in the human genome [10, 11]; however, analysis of the 1000 Genomes Project Data identified many genes under positive selection using the composite method [7, 12]. More recent efforts to identify classic selective sweeps were successful in ascertaining a considerable number of genes under hard selective sweeps [13, 14], using differences in derived allele frequencies (ΔDAF) [15] and two methods that were robust against background selection [16, 17].

A region exhibiting a selective sweep suggests the presence of a local functional variant. This association has been demonstrated via the relationship between gene expression levels and variants showing selection signals [18]. Based on the composite method for the genome-wide scan, the identified regions under selection pressure were suggested to be likely involved in the regulation of gene expression, with a small number of nonsynonymous variants and many variants in the putative regulatory regions [12]. A more recent study evaluated the putative functionality of the population-differentiated regions based on ΔDAF and found that these regions were enriched with genic sites, especially transcription factor-binding sites [13]. Despite the strong evidence that the population-differentiated regions under positive selection are regulatory elements, these recent studies failed to find evidence of relationships between the identified regions and eQTLs, probably because the eQTL analyses were underpowered due to tissue specificity [12].

For the population-specific variants, separate eQTL analyses of each population would fail to identify eQTLs as the derived alleles of target variants are rare in one population and almost fixed in another. Therefore, it is important to perform eQTL analyses in combined populations, assuming that the functional variants affect both populations equally. Accordingly, the patterns in population differences and their functionalities were reinvestigated using whole-genome sequencing data from the 1000 Genomes Project [4]. Two population datasets of African descent in Africa (AFR) and of European descent (EUR) were chosen for the availability of their RNA-sequencing data [19]. These two datasets were examined intensively, and a population of East Asian descent (EAS) was used for comparison. Among the various available estimators of genetic population differences [20–23], the traditional F_ST_ statistic and ΔDAF were respectively chosen in the current study to examine the detailed, region-based population differences at a genome-wide scale and to understand their relationship with functional variants.

## Materials and Methods

### Genome-wide F_ST_ estimations based on sequencing data from the 1000 Genomes Project

Sequencing data from the 1000 Genomes Project were used for estimation of F_ST_. Among various populations, three populations of African ancestry (AFR), East Asian ancestry (EAS), and European ancestry (EUR) were selected for analyses. In AFR, only Africans residing in Africa were included in the current study; therefore, the populations of African ancestry in the Southwest US (ASW) and the African Caribbeans in Barbados (ACB) were excluded. The final numbers were 504 for AFR, 504 for EAS, and 503 for EUR. Whole-genome sequencing data for 22 autosomal chromosomes were examined in the current study.

The Hudson estimator of F_ST_ [24] with bias corrections was used for genome-wide F_ST_ estimations as suggested in the previous study [20]; the ratios of averages were applied to the F_ST_ estimates of a certain genomic range as recommended [20]. Multi-allele variants were divided into multiple variants depending on each allele. For the genome-wide estimates, two different ranges of 1,000 bp and 10,000 bp were used to compare distributions. The start site and the end site were based on the multiples of 1,000 bp or 10,000 bp, and the remnants were excluded from the estimations. Therefore, as shown in Table 1, the number of total variants of the genome-wide estimation based on the 10,000 bp range was smaller than the total number of variants (81,451,971). When examining the derived allele frequencies (DAFs), ancestral alleles that were determined only when all methods provided concordant results were applied from a previous study [25].

**Table 1:** Summary of F_ST_ estimates (ratios of averages) of the total target regions in the human genome.

Index	Coverage	# Variant	F_ST__AFR vs EUR	F_ST__AFR vs EAS	F_ST__EAS vs EUR
**genome-wide**	-	81445891	0.130	0.155	0.098
**repeat**	-	43098205	0.130	0.155	0.098
**non-repeat**	-	38355766	0.131	0.156	0.099
**CDS**	31227897	935636	0.130	0.156	0.099
**5'UTR**	9738399	309216	0.133	0.170	0.104
**3'UTR**	40641568	1188614	0.134	0.156	0.100
**non-coding**	45086573	1443311	0.133	0.159	0.100
**intron**	1212597386	36125625	0.132	0.157	0.100
**-1000**	31163028	929196	0.129	0.156	0.097
**1000**	32474063	980814	0.130	0.155	0.096
**-5000**	94904972	2824410	0.129	0.156	0.098
**5000**	166684490	2511340	0.130	0.155	0.098
**remainder**	1215651307	34205809	0.129	0.153	0.097
**gene region**	1339291823	40002402	0.132	0.157	0.100
**non-genic region**	1540877860	41451569	0.129	0.154	0.097
**sum(shaded)**	2880169683	81453971	-	-	-

The gene-based F_ST_ analyses were performed in two ways: 1) analyses of each gene and 2) whole genome-wide analyses as shown in Table 1. For genome-wide analyses, each region of the 5´ UTR, coding sequences (CDS), 3´ UTR, noncoding gene regions, and introns was examined separately based on ENSEMBL perl API [26]. Additional regions near the gene were also examined: from −5,000 bp to the gene start site, from −1,000 bp to the gene start site, from the gene end site to +1,000 bp, and from the gene end site to +5,000 bp. When there were several transcripts or genes in the same region, priorities were assigned depending on the following order: 1) CDS; 2) 5´ UTR; 3) 3´ UTR; 4) noncoding gene regions; 5) intron; 6) the region between -1,000 bp and the gene start site; 7) the region between +1,000 bp and the gene end site; 8) the region between -5,000 bp and the gene start site; and 9) the region between +5,000 bp and the gene end site. For example, if a genomic region contained an intron of one gene and a 5´ UTR of another gene, the genomic region was considered a 5´ UTR. For repeats and non-repeats, the repeat regions were defined as genomic regions containing at least one repeat sequence, and non-repeat regions as the remainder of genomic sequences containing no repeat sequences.

### Simulations

Simulations were performed based on the forward simulations of the Wright-Fisher model, as reported in previous studies [25]. To examine the differences between the divided populations, the randomly mating original population, with a population size of N, was divided into two populations, each of size N/2. To perform division at the equilibrium state, the original population was randomly mated for 8N generations before division. After division, no migration was assumed and each randomly mating population gradually increased in size from N/2 to N, at a rate of one individual per generation, for N/2 generations. After the restoration of the population size to that of the original population, each population underwent random mating for 4N generations. For the simulations, constant mutation and recombination rates were assumed as 10^−7^/bp per generation. When the population size was 200, a 2,000,000 bp sequence was simulated 10 times in parallel; however, when the population size was 400, a 1,000,000 bp sequence was simulated 20 times in parallel, due to computational limitations.

### eQTL analyses

RNA sequencing data of lymphoblastoid cell lines from a previous study [19] were used for the current analyses. Data were obtained from a total of 465 individuals comprising Utah residents with Northern and Western European ancestry (CEU), Toscani in Italy (TSI), British in England and Scotland (GBR), Finnish in Finland (FIN), and Yoruba in Ibadan, Nigeria (YRI). The final data set of RNA sequencing retained 462 individuals after quantification and normalization [19]. By excluding individuals not found in the Phase 3 sequencing data of the 1000 Genomes Project, eQTL analyses in the current study were conducted for 445 individuals. The previous study divided the samples into Europeans (a total of 373 individuals) and YRI (a total of 89 individuals) to provide two different eQTL results [19]; however, to obtain population-wide functional variants, the current study analyzed all of the samples collectively, thereby providing new results with improved power. In accordance with the previous study [19], variants with a minor allele frequency higher than 0.05 were analyzed.

For eQTL analyses, Matrix eQTL, an R package, was used as in previous studies [19, 27]. Since population-specific expression might exist regardless of the individual genotype, the linear model with a covariate was used to adjust the inherent population specificity [27]. For the regions with the highest F_ST_ estimates, the following four different quantitative traits were analyzed for their relation to genotypes: gene expression, exon expression, repeat expression, and transcription ratios; however, gene expression analyses were conducted for ΔDAF variants, considering the priority and similarity between results for different quantitative traits. For the definition of cis- and trans-eQTL, the default setting of the program [27] was applied, in which cis-eQTL was taken as a significant correlation between a variant and a transcript located within a 10^6^ bp range of the variant position. The cut-off p-value of the trans-eQTL was 0.01, and the cut-off p-value of the cis-eQTL was 0.05. The cut-off for the false discovery rate was 0.05 in the current study.

## Results

### Genome-wide F_ST_ estimation

**Fig 1:**
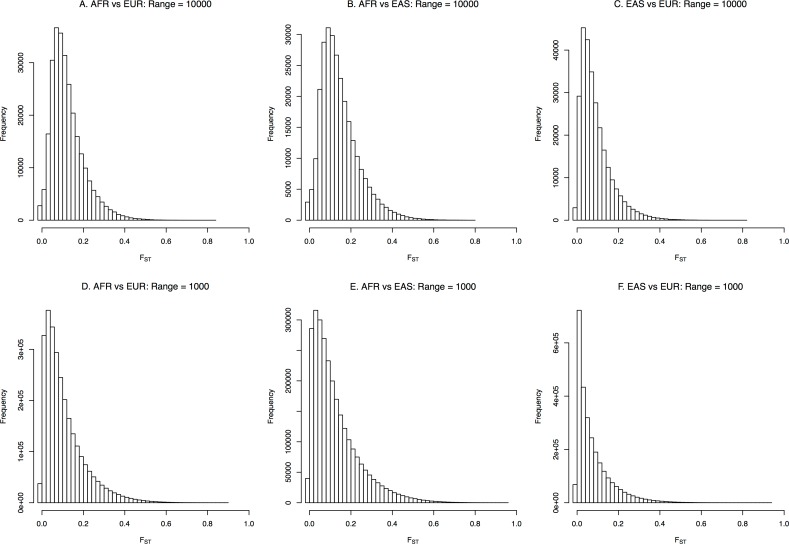
Distribution of genome-wide F_ST_ estimates: (a) AFR vs EUR for the 10,000 bp range; (b) AFR vs EAS for the 10,000 bp range; (c) EAS vs EUR for the 10,000 bp range; (d) AFR vs EUR for the 1,000 bp range; (e) AFR vs EAS for the 1,000 bp range; (f) EAS vs EUR for the 1,000 bp range.

To identify population-specific regions within the genome, the genome-wide F_ST_ estimates were examined in three populations: AFR, EUR, and EAS. Based on a previous study [20], which enabled the estimation of F_ST_ for a certain range, including rare variants, the current study examined the F_ST_ estimates of two different ranges: 10,000 bp and 1,000 bp. As shown in Fig 1, the distribution of F_ST_ estimates differed depending on the range examined. As the estimating range decreased, the distribution of F_ST_ estimates became more dispersed, as smaller numbers of variants in the regions resulted in more diverse F_ST_ estimates. The bias correction term suggested by the previous study [20] resulted in many estimates with negative values (Fig 1), especially for regions with only small numbers of rare variants.

More variants existed in AFR, and the F_ST_ distributions for AFR showed higher degrees of freedom if considered as chi-squared distributions [22]. For the F_ST_ between AFR and EUR, the population-specific regions of the highest 1% of F_ST_ estimates contained many genes (1,220 and 5,730 for the 10,000 bp and 1,000 bp, respectively). However, the number of genes in these regions with the highest 1% of the total F_ST_ estimates was less than the number of genes in the least population-specific region (bottom 1% of total F_ST_ estimates; 1,359 and 11,574 for 10,000 bp and 1,000 bp regions, respectively). These results indicate that genes are preferentially located in the least population-specific regions.

Simulation results (S1 Fig) showed distributions similar to those shown in Fig 1, which appear similar to a chi-squared distribution as previously predicted [22]. The simulations reflected the original population and no sampling of the population was performed; therefore, bias corrections were omitted, providing no F_ST_ estimates below zero. As seen in Fig 1, the F_ST_ distribution was more dispersed as the estimating range became smaller. For the same mutation and recombination rates of 10^−7^, the mean of the total F_ST_ estimates decreased with increasing population size, resulting in slower population differentiation. The means of the F_ST_ estimates were 0.29 and 0.25 for population sizes of 200 and 400, respectively, when the estimating range was 10,000 bp. Similarly, the means were 0.14 and 0.13, respectively, when the range was 1,000. As expected, as the mutation and recombination rates increased, the F_ST_ estimates increased rapidly under the same conditions. The exact relationship between population size and F_ST_ estimates requires further studies.

Considering the importance and variability of structural variants in the genome, the F_ST_ between AFR and EUR was estimated for each structural variant (a total of 38,336) including copy number variants, large deletions or insertions, and inversions as provided by the 1000 Genomes Project. As expected, most structural variants were rare in both populations, and the F_ST_ distribution skewed toward zero. Many structural variants were large enough to involve genes and were therefore likely to be deleterious [28], preventing an increase in their frequency in both populations. The top 1% of F_ST_ structural variants were copy number deletions, which represented the most common structural variant. The total F_ST_ estimate of all structural variants was 0.094. The maximum F_ST_ between AFR and EUR in the current study was 0.508 for a copy number deletion in chromosome 2, harboring *ANKRD36*, followed by 0.494 for a copy number deletion in chromosome 15 harboring *MYO9A*, and 0.472 for a copy number deletion in chromosome 4 harboring *PDLIM3*.

### F_ST_ estimates of genes and repeats

F_ST_ was estimated for each gene in the human genome registered in ENSEMBL [26]. As shown in S2 Fig, the distribution of F_ST_ estimates for noncoding genes was more dispersed than that for the coding genes, with a longer tail, because the sizes of noncoding genes were more diverse with many genes smaller than coding genes. Therefore, the highest F_ST_ estimates of total genes mostly represented noncoding genes rather than coding genes. The highest F_ST_ estimates of noncoding genes were derived from only one or two population-specific variants within the gene region. For the regions of coding and noncoding genes with the highest F_ST_ estimates, the population with the higher allele frequency of the population-specific variants (mostly AFR) usually represented more variants with common frequencies in the extended regions (±5,000 bp of the start and the end sites of the target gene) as shown in S3 Fig. The extended regions also exhibited strong linkage disequilibrium (LD) through D’, and some regions showed strong LD through r^2^ between many variants in the target region (S4 Fig). The data for EAS behaved in accordance to the population with which it shared similarity depending on the population-specific allele examined. DAFs were often almost fixed in the EUR and EAS populations (S5 Fig), similar to a previous study [9].

Eleven of the top 1% of coding genes (a total of 181) were concordant with the gene list (a total of 55) of a previous study [6] based on the F_ST_ estimate of each genotyped variant, which included *SLC24A5*, associated with skin pigmentation, and *EXOC5* and *RNF135*, the 4^th^ and 5^th^ topmost genes, respectively. Many coding genes with the highest F_ST_ estimates were much larger than the topmost noncoding genes, and therefore included numerous population-specific variants within the gene region. For the relatively large genes, as shown in S3B Fig, many population-specific variants showed similar or identical frequencies in the gene region, and these variants were also in strong LD through r^2^ (S4B Fig), suggesting strong selection pressure.

When the overall genome-wide F_ST_ estimates were examined, the gene regions showed higher F_ST_ estimates than the non-genic regions (Table 1), consistent with previous findings [6, 9]. Among the various gene components, the highest F_ST_ estimate for AFR vs. EUR was obtained for the 3´ UTRs and the highest F_ST_ estimates for AFR vs. EAS and EAS vs. EUR were obtained for the 5´ UTRs. In addition, the F_ST_ estimates of the CDS were relatively smaller than those for the total gene regions. These results indicated that the population differences were mostly based on regulation of gene expression rather than on the gene products themselves. However, the regions between ±1,000 bp (or ±5,000 bp) and the gene start (or end) sites showed lower F_ST_ estimates than the gene regions themselves. It is noteworthy that the F_ST_ estimates for EAS were extraordinarily high for the 5´ UTRs, suggesting that the 5´ UTRs of many coding genes were highly differentiated in EAS.

Regions containing repeats undergo evolutionary processes different from those of non-repeat regions. Variants within repeats have been found to experience more rapid evolutionary change than those in non-repeat regions [29, 30]; therefore, F_ST_ estimates of repeat regions might be higher than those of non-repeat regions, if the variants are neutral. However, as shown in Table 1, the total repeat region, throughout the whole genome, showed a slightly smaller F_ST_ estimate of 0.130 compared to the value of 0.131 obtained for the total non-repeat region. The same trend was observed for F_ST_ estimates for other populations, as shown in Table 1. The distribution of F_ST_ estimates also showed a slightly longer tail for the non-repeat regions, although the longer tail resulted from a single high F_ST_ estimate of a specific non-repeat region. The regions of repeats and non-repeats with the highest F_ST_ estimates showed allele frequency distributions, LD plots, and DAF distributions similar to those of genes with the highest F_ST_ estimates.

### eQTL analyses of population-specific genes and variants

The functionalities of regions with the highest and lowest F_ST_ estimates were examined using RNA sequencing data, generated in a previous study [19], for lymphoblastoid cell lines derived from individuals participating in the 1000 Genomes Project. The regions with the highest F_ST_ estimates for four genomic categories, i.e., repeats, non-repeats, noncoding genes, and coding genes, were examined. To examine whether the highest F_ST_ regions were likely eQTLs, the proportions of regions with at least one eQTL, defined by a false discovery rate (FDR) < 0.05, were examined. For coding and noncoding genes, the variants were analyzed in the regions with the highest and lowest 1% of the F_ST_ estimates; for repeat and non-repeat regions, the variants were analyzed in the regions with the highest and lowest 100 F_ST_ estimates.

**Fig 2:**
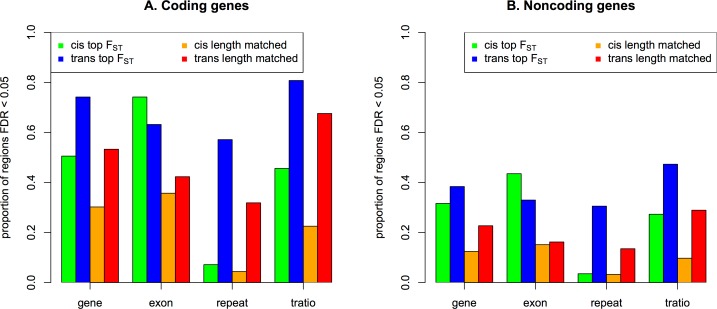
Proportions of regions having eQTLs with FDR < 0.05 among the total regions analyzed.

The highest F_ST_ regions contained overwhelming proportions of eQTL regions compared to the lowest F_ST_ regions (S6 Fig), most of which contained only rare variants. Because the eQTL analyses were conducted for variants with minor allele frequencies > 0.05, most of the regions with the lowest F_ST_ did not consist of variants to be analyzed, as indicated previously [13]. Even when only the regions with the lowest F_ST_ that had variants for eQTL analyses were included, the trend that the regions with the highest F_ST_ were still more likely to consist of eQTLs did not change, except for a few repeat cis-eQTLs. Repeats had trends that were similar to those of non-repeats, and coding genes had trends that were similar to those of noncoding genes, with a higher proportion of exon cis-eQTLs than exon trans-eQTLs. Accepting the concerns described previously [13], the regions with lengths that were matched to the genes with the highest 1% of F_ST_ genes were examined as shown in Fig 2. The regions with the highest F_ST_ clearly were more likely to be eQTL when compared to the regions with matched lengths, showing significant p-values through simple chi-squared tests (most p-values < 0.001), except for the proportions of repeat cis-eQTLs. These results indicated that the genes with the top F_ST_ likely included eQTLs.

For the top ten regions, many population-specific variants were classified as eQTL with FDR < 0.05 (S1 Table); many were also both cis- and trans-eQTLs. Notably, population-specific variants in repeats or non-repeats with the highest F_st_ estimates tended to regulate repeat expression rather than gene expression, and noncoding and coding genes tended to regulate gene rather than repeat expression. Several population-specific variants in repeats and non-repeats were found to be eQTL hotspots, regulating many genes simultaneously (S1 Table). Several population-specific variants in noncoding genes were also eQTL hotspots, and most of the population-specific coding genes were strong cis- and trans-eQTLs (S1 Table). Especially, most population-specific variants with similar allele frequencies in these genes were also identified as eQTLs, which substantially increased the number of eQTLs with FDR <0.05. The evidence of strong population-specific regulator variants suggested the importance of these genes in determining the phenotypic differences between AFR and EUR.

**Fig 3:**
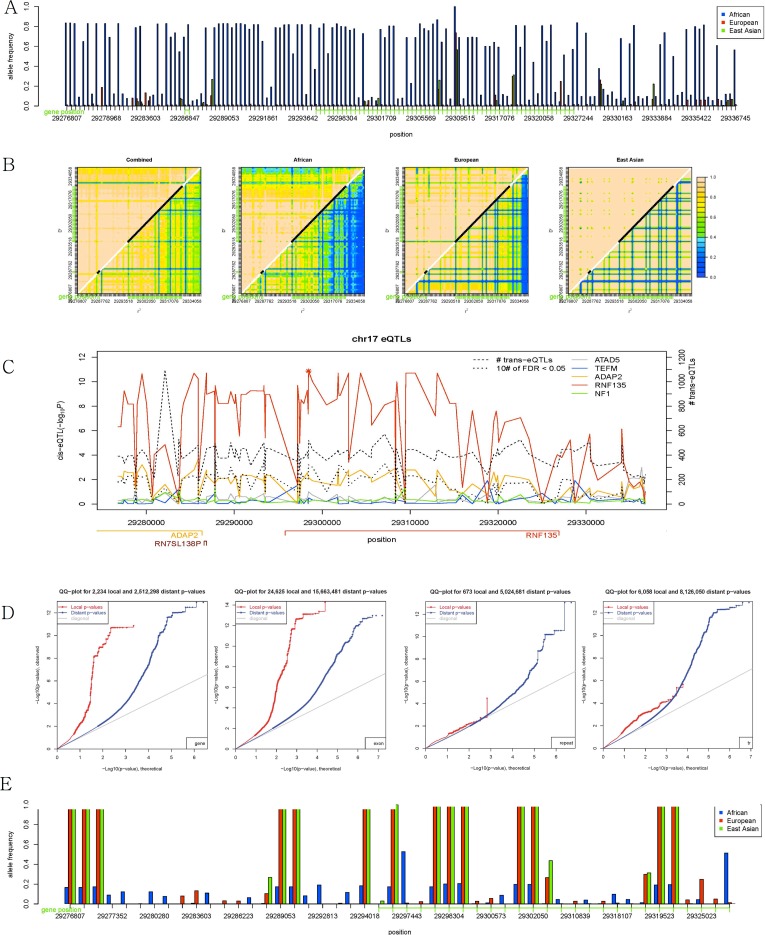
Extended regions from the population-specific regions between AFR and EUR on chromosome 17: (a) Allele frequency distribution; (b) linkage disequilibrium; (c) expression QTL; (d) quantile-quantile plot of cis- and trans-eQTL; (e) derived allele frequency distribution.

Among the population-specific genes that were also eQTLs, one coding gene (*RNF135*) and one noncoding gene (*RN7SL138P*) were located close to each other on the *NF1* microdeletion region of chromosome 17 [31]. The extended region from –10,000 bp from the start site of *RN7SL138P* to +10,000 bp from the end site of *RNF135* was therefore examined in depth (Fig 3). Consistent with the findings for allele frequency distributions of the extended regions of each gene, more variants with high frequencies were observed in AFR than in EUR (Fig 3A), possibly due to the reference genome. The entire region was in strong LD through both D’ and r^2^, as shown in Fig 3B; however, the LD was strongest in EAS and stronger in EUR than in AFR. As shown in Fig 3C, most population-specific variants in this region regulated the expression of *RNF135*, indicating that the strong cis-eQTL resulted from this regulation. Furthermore, as shown in Fig 3D, most variants in strong LD with each other in the region were also strong trans-regulators. Therefore, in this region, the gene product of *RNF135* most likely acted as a trans-regulator to control the expression of many other genes at distant loci via a cis activation. To summarize, one or several population-specific variants in this region regulate the expression of *RNF135*, and, in turn, the gene product of *RNF135* regulates other genes at distant loci.

One variant (rs113617171) in the region showed an extraordinarily large number of trans-eQTLs (Fig 3C); however, the number of eQTLs with FDR <0.05 was not high compared with other variants. Most gene enrichment analyses using gene ontology failed to produce a meaningful biological process that was enriched in the gene lists; however, the gene list obtained from rs113617171 showed significant p-values for the following enriched biological processes [32, 33]: RNA splicing (1.8 × 10^−2^), chromatin organization (2.3 × 10^−4^), organelle organization (6.14 × 10^−4^), cellular component organization (2.48 × 10^−2^), protein metabolic process (5.89 × 10^−3^), metabolic process (9.66 × 10^−8^), and primary metabolic process (3.04 × 10^−5^), in the order of fold enrichment. The biological processes that showed fewer numbers of expected genes were as follows: developmental process (3.82 × 10^−2^), system development (2.98 × 10^−4^), ectoderm development (1.42 × 10^−2^), and nervous system development (5.27 × 10^−5^). The Bonferroni correction was applied to all of the results.

As shown in Fig 3E, most of the high-frequency alleles in AFR shown in Fig 3A were ancestral alleles, and many derived alleles were almost fixed in EAS and EUR; both EAS and EUR contained small numbers of common variants. This result suggests a recent selective sweep in the region of chromosome 17 harboring *RN7SL138P* and *RNF135*, which was also a strong cis- and trans-regulator as described above. As previously suggested [18], a bottleneck and exposure to new environment could explain the reason why EAS and EUR were primarily affected by the selective sweep representing extreme population differences in the region; however, the allele frequency distribution and strong LD indicated the possibility that AFR had also experienced selection pressure. A recent study found that a missense variant (rs111902263) in *RNF135* was associated with autism [34]; however, although the accuracy problem of detecting ancestral alleles exists, the derived allele frequencies of the variant were very low (0.002, 0.027, and 0 for AFR, EUR, and EAS, respectively), indicating this variant might not be the driver.

### Relationship between ΔDAF and eQTLs

The F_ST_ estimates of certain regions were dependent on the length of the estimated regions based on simulation studies (S1 Fig); therefore, the attempts to determine an overall connection between eQTLs and population differentiation based on gene-based F_ST_ estimates results in a false relationship due to the intrinsic dependency of eQTLs on the lengths. However, single-variant analyses could provide no such correlation regarding the impact of functional variants on population differentiation. The F_ST_ estimate of each single variant could be applicable; however, for simplicity, ΔDAF was examined in the current study, especially for the ΔDAFs of all variants in coding and noncoding genes. As variants with minor allele frequencies higher than 0.05 in the data were included in the eQTL analyses, the ΔDAFs of these variants were examined. In total, 2,895,539 variants in coding genes and 1,071,250 variants in noncoding genes were included.

**Fig 4:**
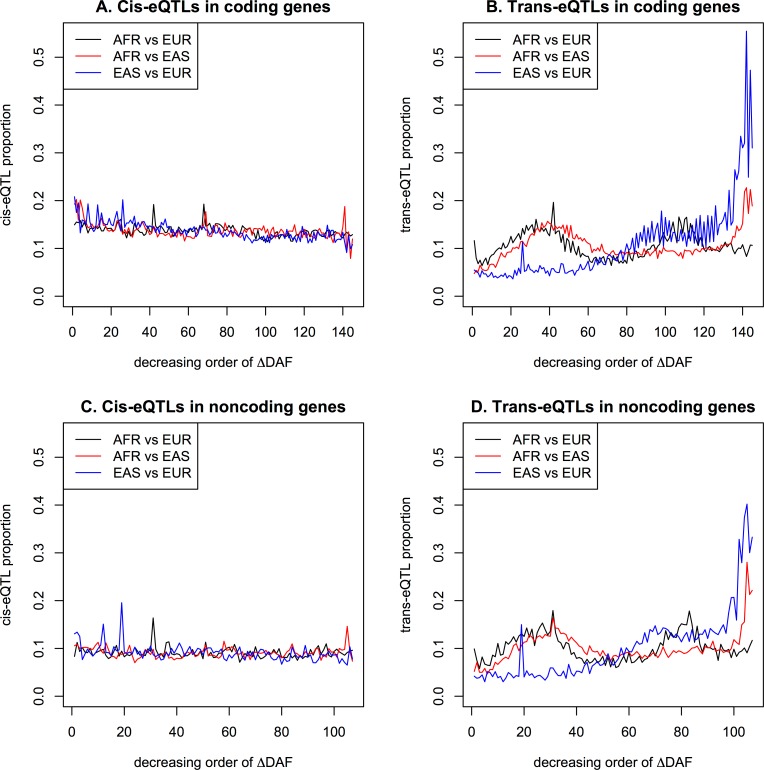
Proportions of eQTLs in groups with decreasing ΔDAF: (a) cis-eQTLs in coding genes; (b) trans-eQTLs in coding genes; (c) cis-eQTLs in noncoding genes; (d) trans-eQTLs in noncoding genes.

To examine the overall relationship between population differentiation and functionality, the variants were ordered and grouped based on ΔDAF, with each group consisting of 20,000 or 10,000 variants depending on the decreasing order of ΔDAF for coding or noncoding genes respectively. Therefore, there were 145 groups for 2,895,539 coding variants and 107 groups for 1,071,250 noncoding variants. The proportions of variants having eQTLs with FDR < 0.05 in each group were plotted, as shown in Fig 4. Assuming no influence of the eQTLs on population differentiation, random yet constant proportions were expected as shown in Fig 4A and 4C for cis-eQTLs, although a slight tendency to decrease was observed, especially in coding genes in this study. As shown in Fig 4B and 4D for trans-eQTLs, high proportions in the groups with high ΔDAF between AFR and EUR (or EAS) indicated selection pressure acting on a specific population, and high proportions in the groups with low ΔDAF, specifically between EAS and EUR, indicated global selection pressure on both populations. The patterns of eQTL proportions grouped depending on the ΔDAF between AFR and EUR were similar to those between AFR and EAS and different from those between EAS and EUR. The patterns were very similar between coding and noncoding genes, but differed between cis-eQTLs and trans-eQTLs. The spikes in Fig 4 were usually derived from one region containing many variants with similar DAFs, possibly due to selection pressures on broad regions.

**Fig 5:**
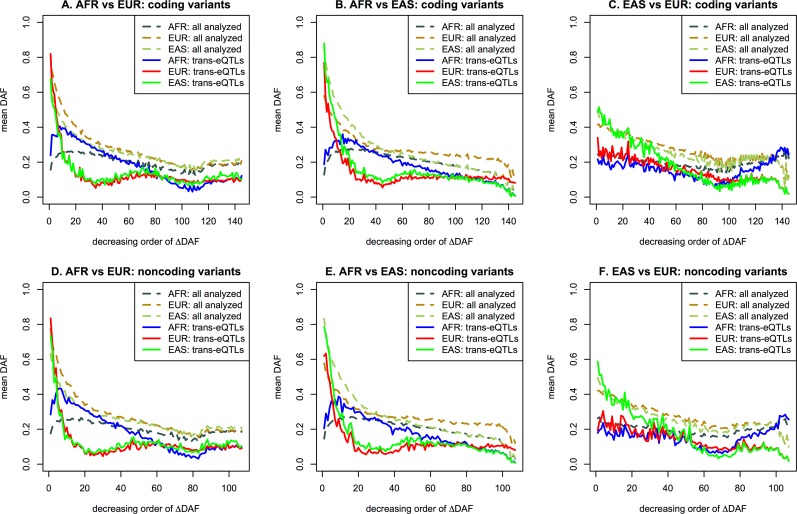
Mean DAFs in groups with decreasing ΔDAF: (a) AFR vs EUR: coding genes; (b) AFR vs EAS: coding genes; (c) EAS vs EUR: coding genes; (d) AFR vs EUR: noncoding genes; (e) AFR vs EAS: noncoding genes; (f) EAS vs EUR: noncoding genes.

The mean DAFs of functional variants depending on the ΔDAF groups showed larger mean DAFs in EAS and EUR among the most highly differentiated groups in Figs 5 and S7, indicating strong recent adaptations in these two populations. The same trends were observed in all plots. The mean DAFs of cis-eQTLs showed patterns similar to those of all variants, in which the differences between population groups reduced as ΔDAF decreased for cis-eQTLs. For the trans-eQTLs in Fig 4, the groups with high, yet not the highest, ΔDAF between AFR and EUR (or EAS) showed high proportions, indicating either more recent or weaker adaptation. Fig 5 showed a larger mean DAF for AFR than for EAS and EUR in the groups with high, yet not the highest, ΔDAF, suggesting that such more recent or slower adaptation occurred specifically in AFR.

Notably, in Fig 4, the groups with the lowest ΔDAF between EAS and EUR showed very high proportions of trans-eQTLs, indicating common selective pressures in both EAS and EUR. The selective pressures were likely negative rather than positive because the mean DAF of EAS and EUR was low for the corresponding low ΔDAF groups between EAS and EUR (Fig 5C and 5F). Here, because the mean DAF of AFR was higher than the overall DAFs as well as the mean DAF of both EAS and EUR, the negative selection of the corresponding functional variants in EAS and EUR looks slightly positive or at least neutral in AFR. The slightly higher trans-eQTL proportions shown in Fig 4B and 4D in groups with the low, yet not the lowest, ΔDAF between AFR and EUR indicated common negative selective pressures on trans-eQTLs strongly in AFR and less strongly in EUR as shown in Fig 5A and 5D.

**Fig 6:**
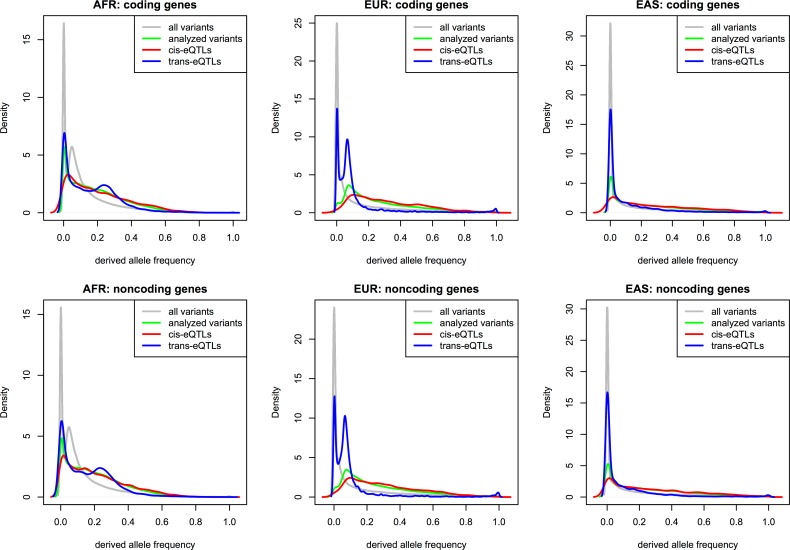
Distributions of DAFs of all variants, analyzed variants, cis-eQTLs, and trans-eQTLs in gene regions.

For neutral variants, the distribution of DAFs exhibited a very high density near zero, followed by rapid continuous decrement. Notably, the DAFs for all frequency variants in the gene regions showed unusual distributions (Figs 6 and S8). The DAF distributions of all three populations showed a slight bump near one, indicating the existence of functional variants under selective pressure close to fixation. The phenomena can be observed with false identifications of ancestral alleles [25]; however, because the ancestral identifications based on the concordance of all three methods did not produce the phenomena in the previous study, the DAF distributions for gene regions clearly presented the existence of functional variants close to fixation. All three populations also showed a bump at specific points prior to 0.01 that differed depending on population. AFR showed the most distinctive peak, indicating many variants under ongoing selection pressure, whereas EAS showed a gentle slope almost buried at the starting peak. Overall, for all populations, the observations indicated that variants under selection pressure existed in gene regions, some of which were close to fixation, and others under very recent or weak selective pressure.

## Discussion

The unusual patterns of allele frequencies and strong LD of the topmost differentiated regions supported the existence of selective sweeps, as has been recently suggested [12, 14]. More importantly, the intricate relationship between the grouped ΔDAF and eQTLs conclusively indicated the complexities of recent human adaptation, mainly via regulatory manipulations. When the top 1% of coding genes in the current study were compared with the genes located in the top 1% of regions under classic selective sweeps in a previous study [14], a few genes common to both lists were identified. The previous study did not highlight *RNF135* on chromosome 17 as a region of selective sweep, although this gene was included in the gene list under strong selection pressure in another study by the same group [6]. Additionally, no genes of ancient selective sweeps from another recent study [35] were found in the list of the highest 1% of coding genes in the current study, indicating that the selection pressure on the highly differentiated genes was possibly quite recent.

The method based on long-range haplotype did not find this *RNF135* region under selection pressure [36]. However, 22 genes under positive selection discovered using the composite method were coincident with the highest 1% of coding genes [12]. Among the top ten coding genes in the current study, five genes (including *RNF135*) were also listed in the identified region using the composite method; however, *RNF135* was found to be under selection pressure only in the population from Yoruba in Ibadan, Nigeria (YRI) in this previous study [12], although stronger selective sweeps occurred in EAS and EUR than in AFR, as shown in Fig 3E. Only nine population-differentiated regions from the recent study [13] were coincident with the highest 1% of coding genes, including *CTXN2* and *ZMYM6*, among the top ten coding genes. This discordance again indicated the difficulty in detecting selection pressure [37], and improvements in identifying regions under selection pressure might be useful to resolve the remaining problems.

The F_ST_ estimator suggested in a previous study [20] showed reliable results in finding the population-specific regions of the human genome. However, the bias correction terms in the equation [20] led to unrealistic F_ST_ distributions with many negative estimates. Although the bias term approaches zero as the sample sizes increases, it may be preferable to reconsider the use of an unbiased F_ST_ estimator, especially when the distribution of F_ST_, or small regions, is of interest. The distributions of F_ST_ estimates differed depending on the range examined, when the simulations were performed assuming genetic drift for neutral regions. As the estimating range decreased, the smaller numbers of variants in the regions resulted in more diverse F_ST_ estimates and finally more dispersed distribution of F_ST_ estimates. Therefore, smaller genes were observed more frequently than larger genes in the groups with the highest and lowest F_ST_ estimates, and larger genes were mostly observed in the group with mid-range F_ST_ estimates. When the proportions of large genes (> 10,000 bp in size) in F_ST_ groups were plotted similarly to Fig 4, a slight skew towards groups with high F_ST_ was observed. Large population-specific genes under strong selective sweeps would harbor many population-specific variants with similar allele frequencies under strong LD, possibly representing overlapping eQTL signals.

There have been concerns on the use of F_ST_ regarding their dependency on diversity within a population, and these concerns have lead to multiple other estimates that are also applicable to multi-allelic variants [38–41]. The concern was mainly due to the highly mutable and highly variable sites such as microsatellites. In the current study, the effect of within population diversity might not be critical because the main focus was on nucleotide subsitutions and insertions/deletions. Similarly, the V_ST_ statistic, which is applicable to multi-allelic variants [42], was applied to structural variants [43]. The V_ST_ statistic is specialized for structural variants based on the variance of the log_2_ ratios in the identification of structural variants [42]. However, when identifying population-specific structural variants, each population-specific allele could be of interest as opposed to examining all of the alleles within the multi-allele variants. Therefore, the current study utilized the traditional F_ST_ statistic rather than other statistics. Furthermore, structural variants exhibited smaller F_ST_ estimates than other types of variants, which suggested that structural variants might not play a crucial role in population-specific phenotypes.

Regarding the DAF distributions for eQTLs, because the constitution of populations for eQTL variants involved mostly EUR, the DAF distributions were biased as shown in S8 Fig; this was also reflected in the DAF distributions of the eQTLs. Regardless of the intrinsic distortions due to confinement, the DAF distributions of eQTLs showed distinctive features compared to the DAF distributions of analyzed variants, as shown in Fig 6. For EAS and EUR, the DAF distributions of the trans-eQTLs showed clear bumps near one, indicating almost fixed functional variants. For all populations, the DAF distributions of the cis-eQTLs showed slightly higher densities for the DAFs of common variants, indicating cis-regulating variants under selective pressure, which was also coincident with the slight negative correlations between groups with decreasing ΔDAF and the proportions of cis-eQTLs in Fig 4. In comparison with the unusual distribution of all DAFs in gene regions, as shown in Fig 6 and S8 Fig, the DAF distributions of eQTLs likely indicate the existence of selection pressure on functional variants other than those underlying the eQTLs of the lymphoblastoid cell lines.

In the current study, the eQTL analyses were based on RNA sequencing of lymphoblastoid cell lines. The population-specific variants might show different results depending on the tissue examined due to tissue specificity [44]. Recent epigenome analyses have demonstrated that population differences are not confined to the genome [45], identifying regions wherein methylation patterns differed between populations in epidermis samples. It is questionable whether the differences arose from genetic differences, environment, or inherited methylation patterns. Further studies could elucidate the exact mechanisms underlying complex traits and their population differences in consideration of tissue specificity and various omics data.

Differential demographic changes in populations affect the allele frequency distributions, which also can influence the population differentiation estimates [46]. The influence of the most recent demography is the largest, and the recent expansions have been observed for most human populations [4, 47]. Therefore, the demographic influence could be similar in all studied populations. In addition, population structures exist even in populations in regions that are within close geographical proximity, which can influence any population-based associations [48, 49]. The current study employed the regional populations, including only individuals who had been residing in the region so as to minimize the effects of migration or population structure. The method of detecting selection pressures based on individuals rather than populations did not identify the *RNF135* region as the region under strong selection pressures, although the method is free from the false positives due to the population structure [48]. Removing these effects of demographic changes and population structure could be a solution, as provided previously [50].

Beyond genome-wide genotyping [1] and whole-genome sequencing [2], the functionality of genetic regions or variants has received attention for better understanding the genetic architecture of complex traits [19]. However, the contribution of rare variants to the manifestation of complex traits [51–53] makes the identification of rare functional variants and understanding of their underlying mechanisms challenging. Rare variants in one population may occasionally be common in another, and genetic differences between populations may provide valuable insights into the influence of rare functional variants. Furthermore, the elucidation of population differences is essential for realizing the promise of precision medicine, which is aimed at providing customized prevention and treatment to each individual [54].

To establish evidence of the functional roles of population differentiation, genome-wide F_ST_ estimations, ΔDAFs and eQTL analyses of all variants in the gene regions were conducted in the current study. The suggested F_ST_ statistic was useful for examining regions under population differentiation, and the eQTL analyses of highly differentiated regions indicated the importance of regulatory manipulations in population differentiation. To examine the relationship between functionality and population differentiation, ΔDAFs of whole variants in gene regions were grouped depending on their ΔDAFs. The analyses of ΔDAF groups showed complicated patterns of recent human adaptation through gene regulation, including strong adaptation common to EAS and EUR as well as mild or more recent adaptation in AFR. The current study provides the first actual evidence of intricate recent human adaptations. Additional articulated studies would be helpful to identify and understand recent human adaptation.

## Supporting Information

S1 Fig Simulation results of F_ST_ estimates for two populations after the division at N/2+ 4N generations ago: (a) Distribution of F_ST_ estimates when N was 200 and the estimating range was 10,000 bp; (b) Distribution of F_ST_ estimates when N was 200 and the estimating range was 1,000 bp; (c) Distribution of F_ST_ estimates when N was 400 and the estimating range was 10,000 bp; (d) Distribution of F_ST_ estimates when N was 400 and the estimating range was 1,000 bp.
(PDF)

S2 Fig Distribution of F_ST_ estimates between AFR and EUR: (a) Coding genes; (b) Non-coding genes.
(PDF)

S3 Fig Allele frequency distribution of the extended regions (±5,000) of the top F_ST_ estimates of coding and noncoding genes; (a) Noncoding genes; (b) Coding genes.
(PDF)

S4 Fig Linkage disequilibrium in the extended regions (±5,000) of the top F_ST_ estimates of coding and noncoding genes for the combined population: AFR, EUR, and EAS; (a) Noncoding genes; (b) Coding genes.
(PDF)

S5 Fig Derived allele frequency distribution of the extended regions (±5,000) of the top F_ST_ estimates of coding and noncoding genes; (a) Noncoding genes; (b) Coding genes.
(PDF)

S6 Fig Proportions of regions having eQTLs with FDR < 0.05 among the total regions for top 1% and bottom 1% of F_ST_ estimates. (PDF)

S7 Fig Mean DAFs depending on the decreasing ΔDAF groups. (PDF)

S8 Fig Distributions of DAFs of all variants and analyzed variants in gene regions. (PDF)

S1 File The raw data tables: comRSgenes.txt & comRiSgenes.txt: top and bottom F_ST_ estimates with the estimating range of 10,000 bp; comRSgenes1000.txt & comRiSgenes1000.txt: top and bottom F_ST_ estimates with the estimating range of 1000 bp; cisdafC.csv & transdafC.csv: cis and trans eQTLs in coding genes with FDR <0.05; cisdafN.csv & transdafN.csv: cis and trans eQTLs in noncoding genes with FDR <0.05; and etc. (ZIP)

S1 Table Summary of the number of eQTL that passed FDR <0.05. (DOC)
