# Supplementary figures and images for: Evidence of Recent Intricate Adaptation in Human Populations
Source: PLoS One. 2016 Dec 19;11(12):e0165870. doi: 10.1371/journal.pone.0165870 (PMC5167553; doi:10.1371/journal.pone.0165870)

A.  $N=200$   $SN=10000$   $F_{ST}$

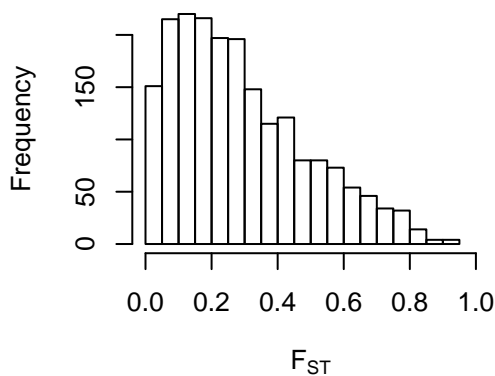

B.  $N=200$   $SN=1000$   $F_{ST}$

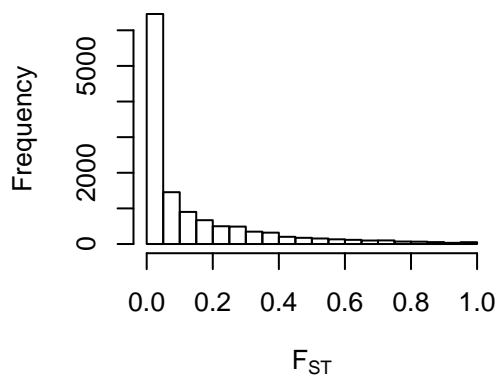

C.  $N=400$   $SN=10000$   $F_{ST}$

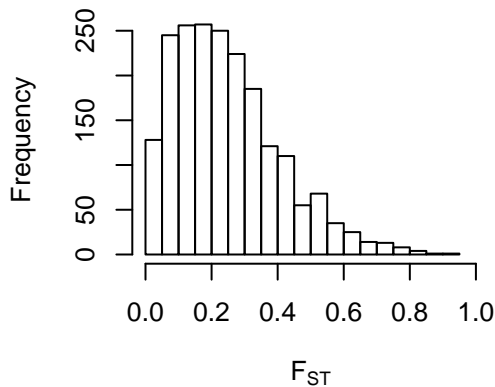

D.  $N=400$   $SN=1000$   $F_{ST}$

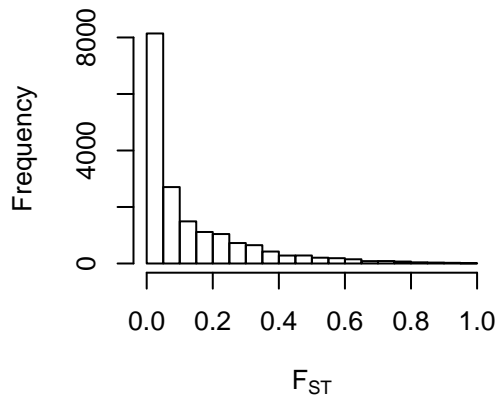

Supplement: S1 Fig — Simulation results of FST estimates for two populations after the division at N/2+ 4N generations ago: (a) Distribution of FST estimates when N was 200 and the estimating range was 10,000 bp; (b) Distribution of FST estimates when N was 200 and the estimating range was 1,000 bp; (c) Distribution of FST estimates when N was 400 and the estimating range was 10,000 bp; (d) Distribution of FST estimates when N was 400 and the estimating range was 1,000 bp. (PDF) [file pone.0165870.s001.pdf]

A.  $F_{ST}$  of 18157 coding genes

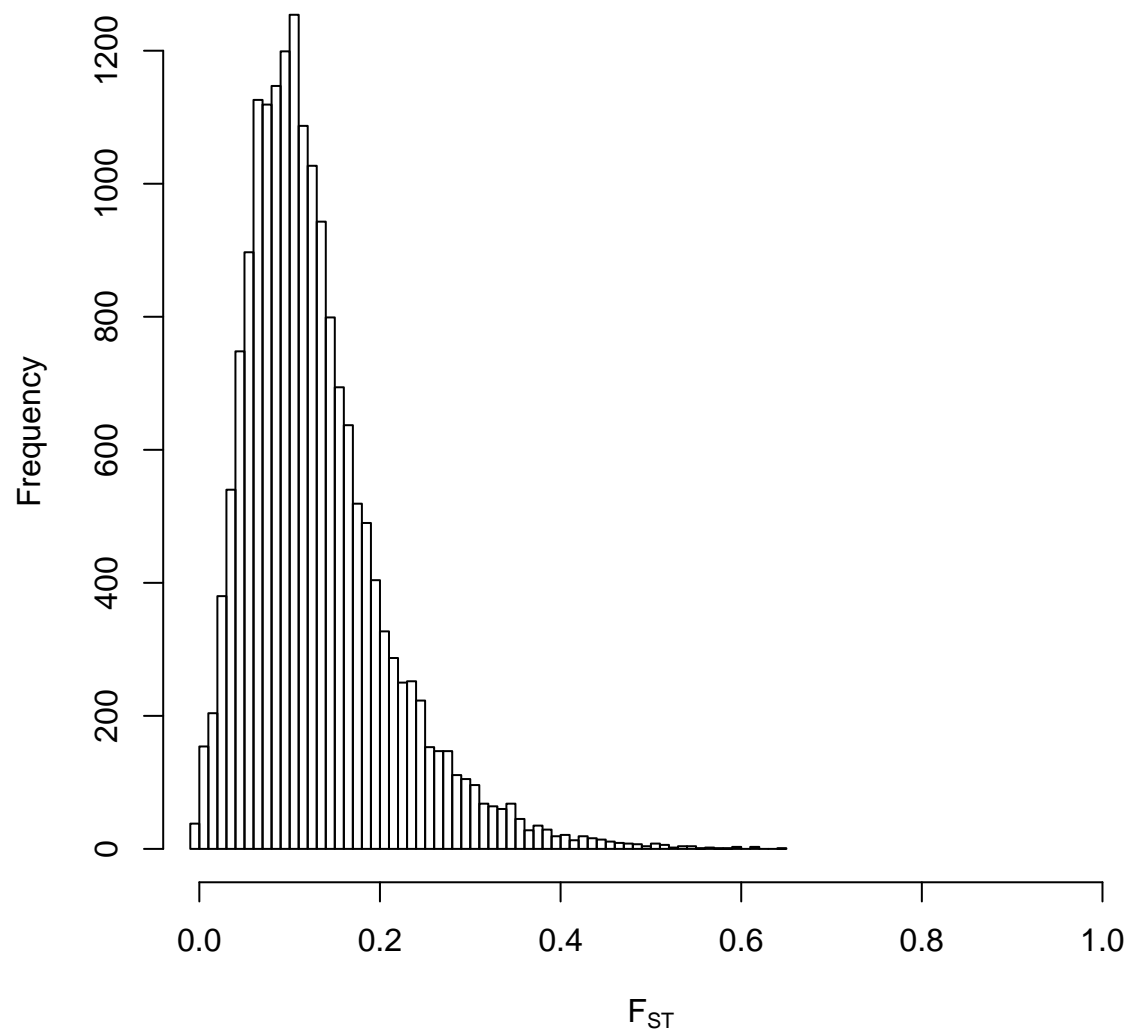

B.  $F_{ST}$  of 37048 noncoding genes

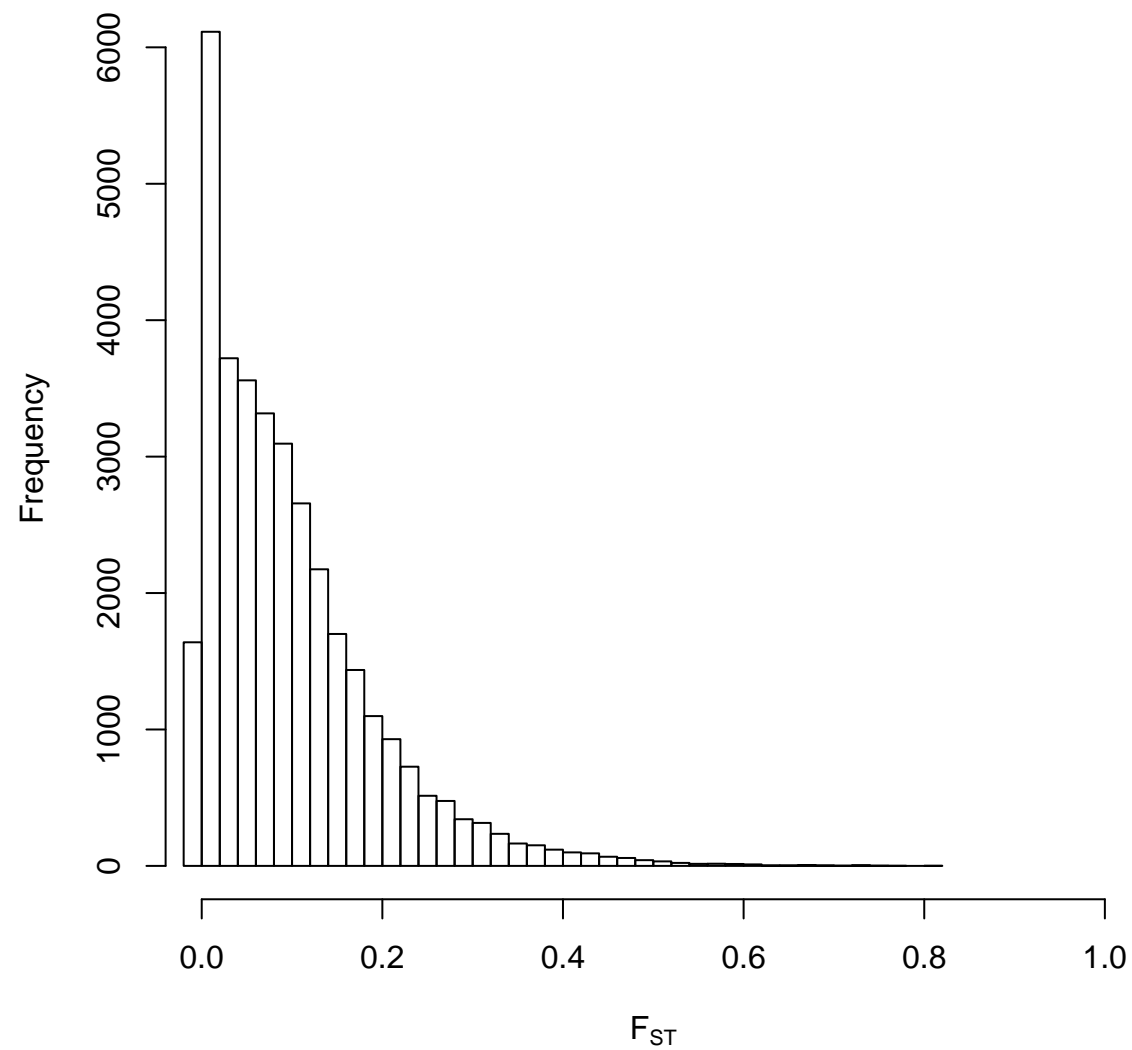

Supplement: S2 Fig — Distribution of FST estimates between AFR and EUR: (a) Coding genes; (b) Non-coding genes. (PDF) [file pone.0165870.s002.pdf]

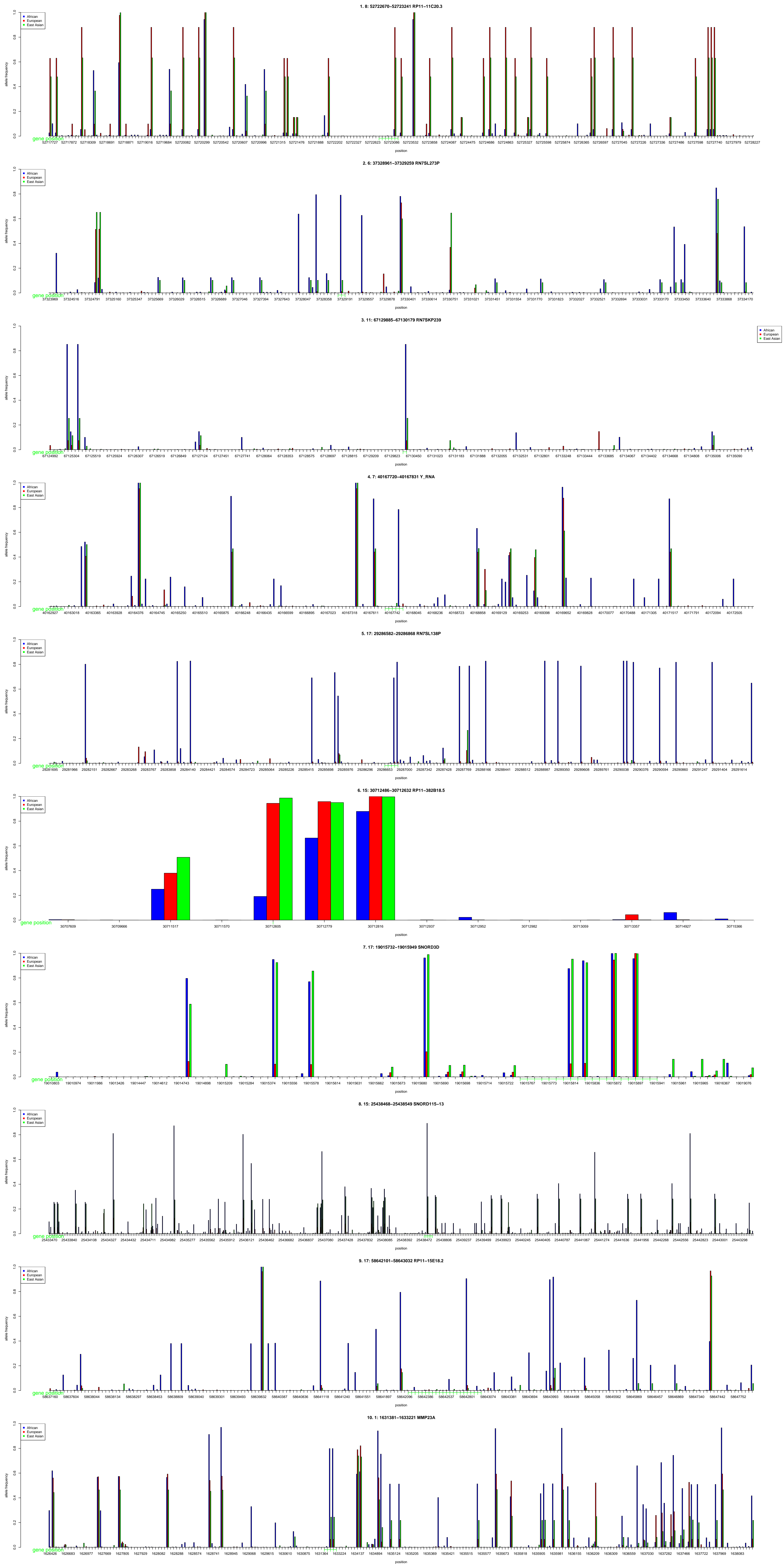

1. 16: 14841923-14859270 NPIPA2

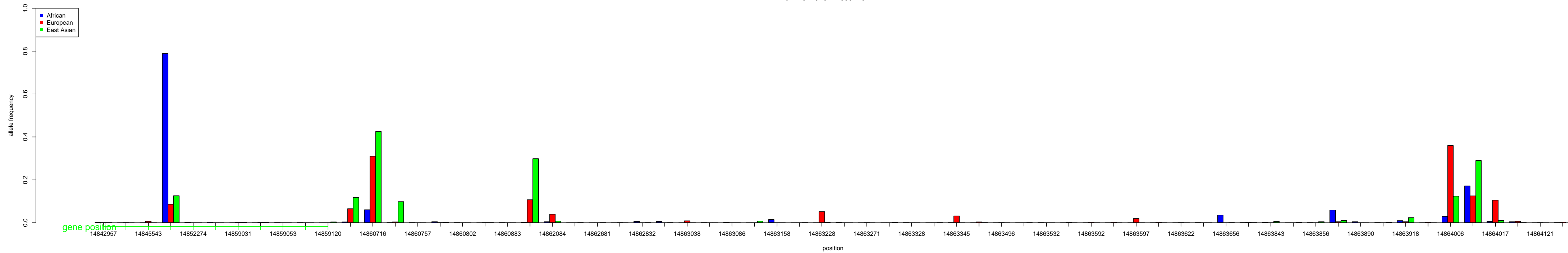

2. 2: 74682150-74685087 INO80B

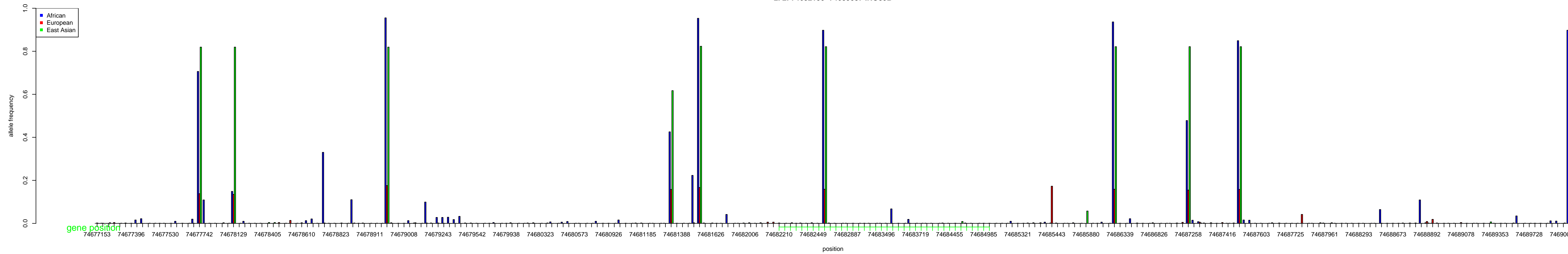

3. 8: 52730135-52811746 PCMTD1

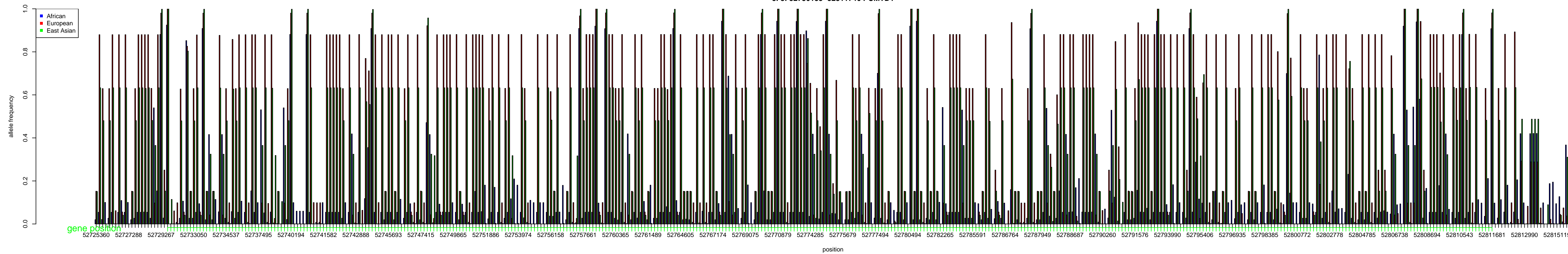

4. 14: 57667225-57735726 EXOC5

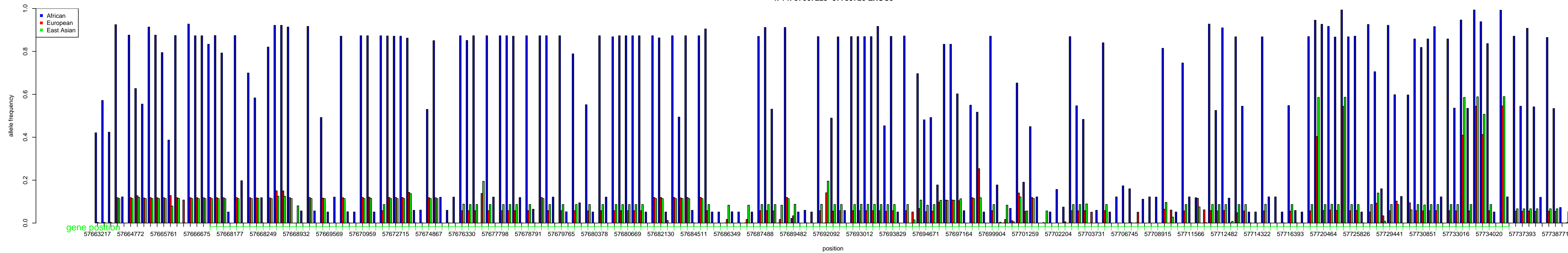

5. 17: 29295803-29326929 RNF135

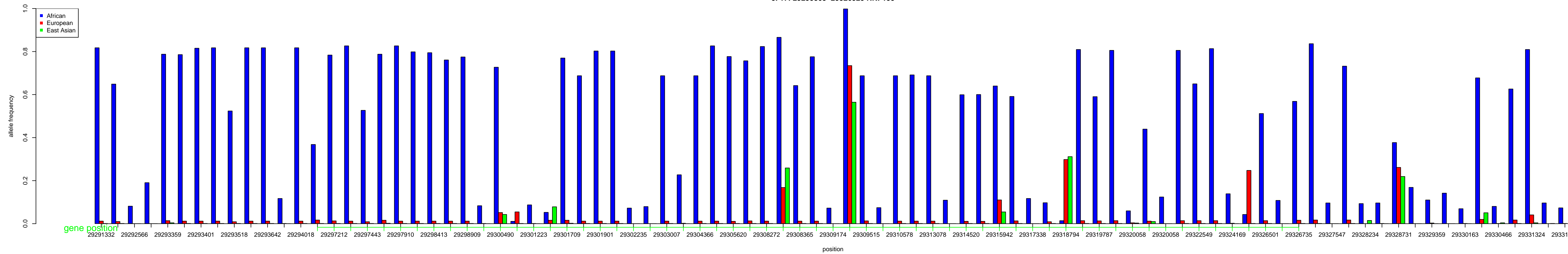

6. 7: 38331217-38331679 TRGV11

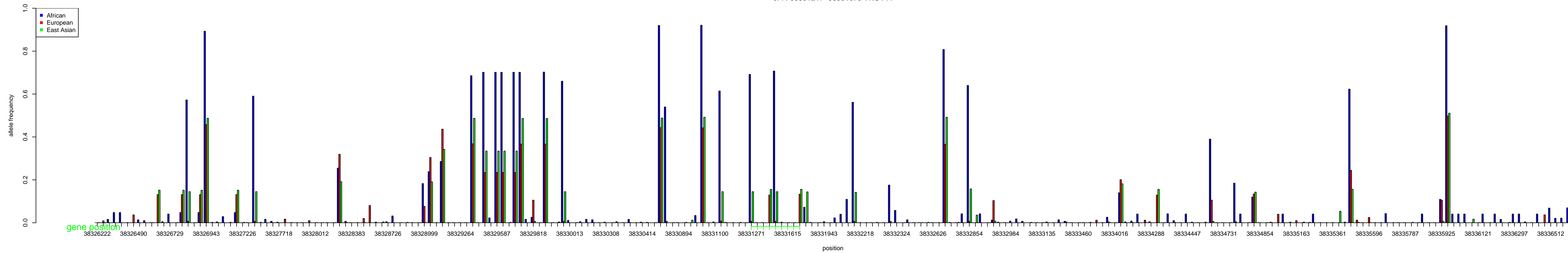

7. 14: 106053226-106054732 IGHA2

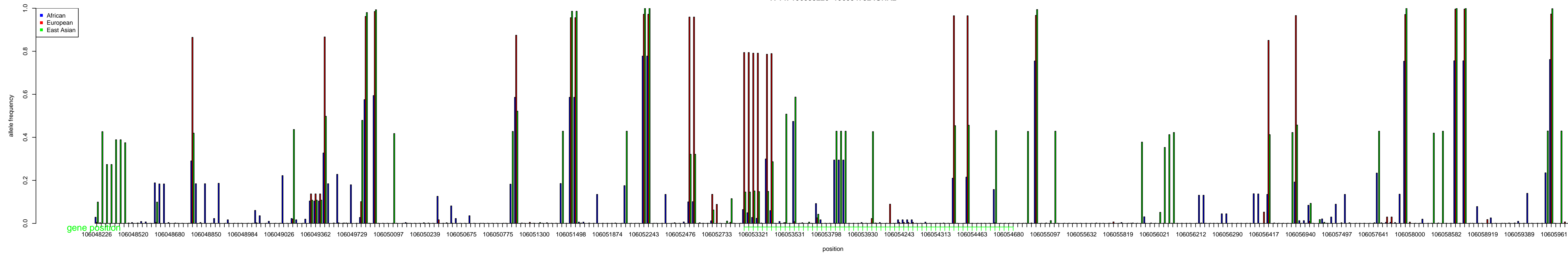

8. 15: 48483736-48495953 CTXN2

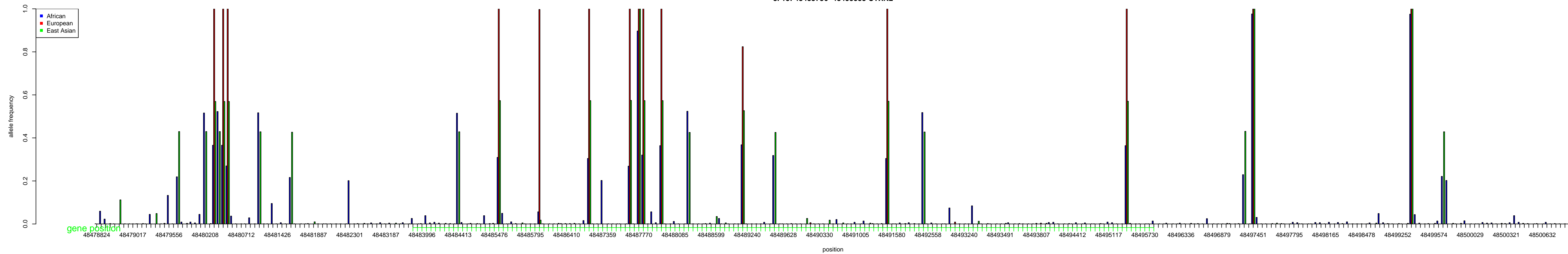

9. 1: 53519296-53520621 AL445183.1

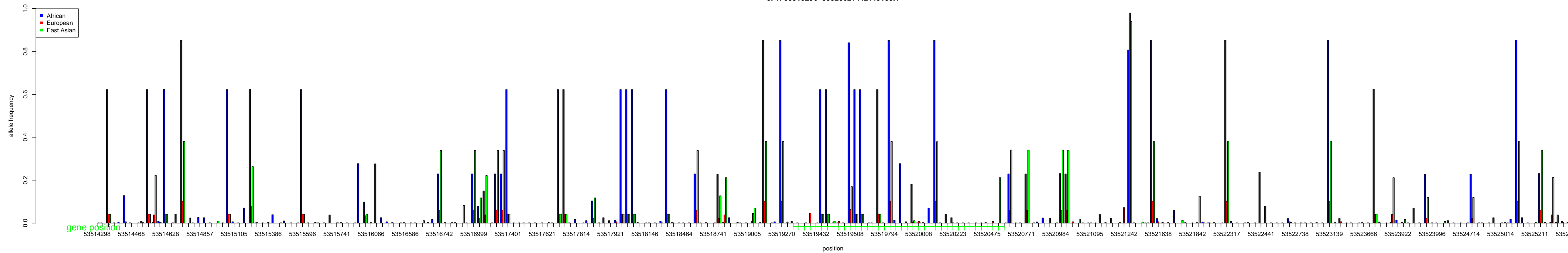

10. 1: 35451766-35497569 ZMYM6

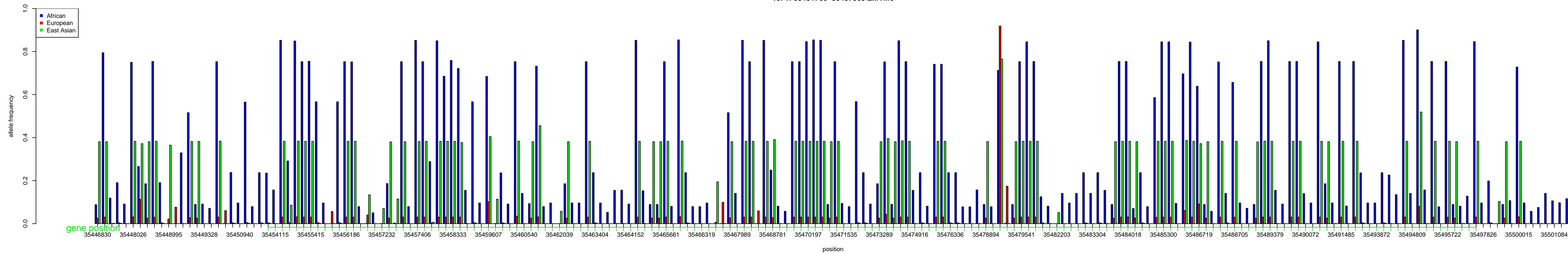

Supplement: S3 Fig — Allele frequency distribution of the extended regions (±5,000) of the top FST estimates of coding and noncoding genes; (a) Noncoding genes; (b) Coding genes. (PDF) [file pone.0165870.s003.pdf]

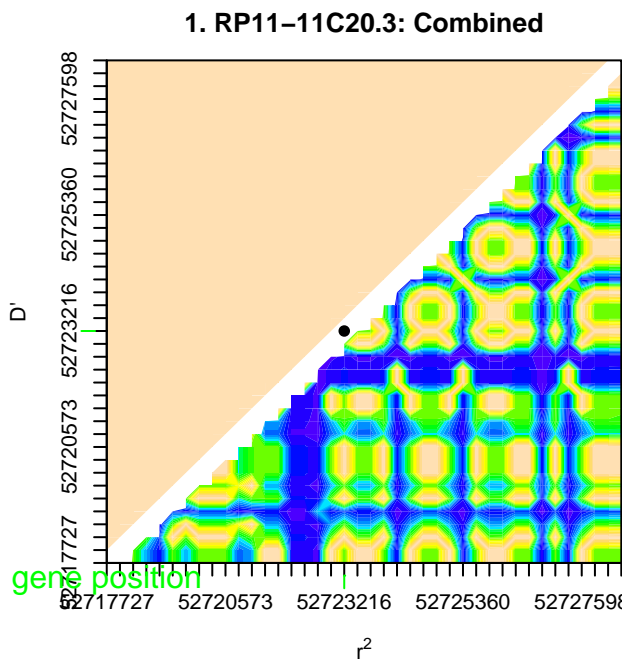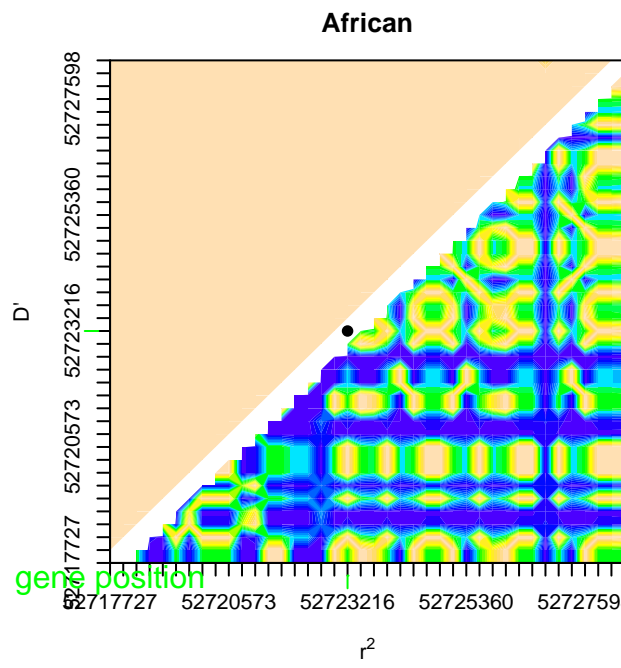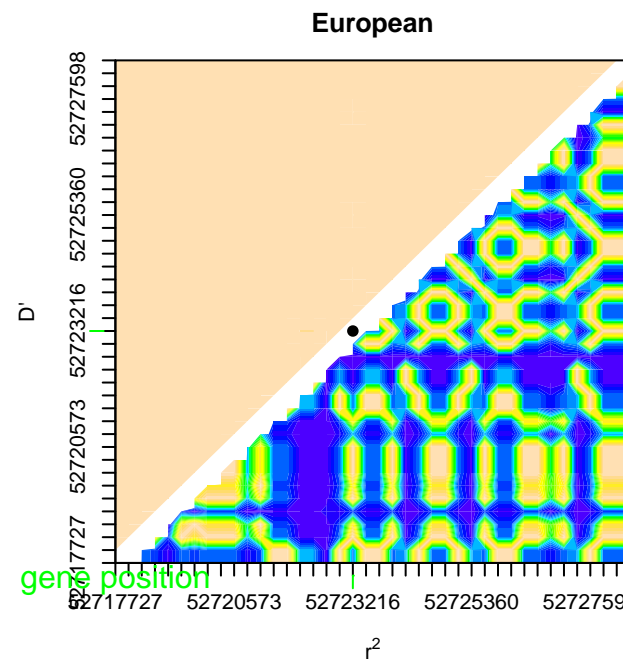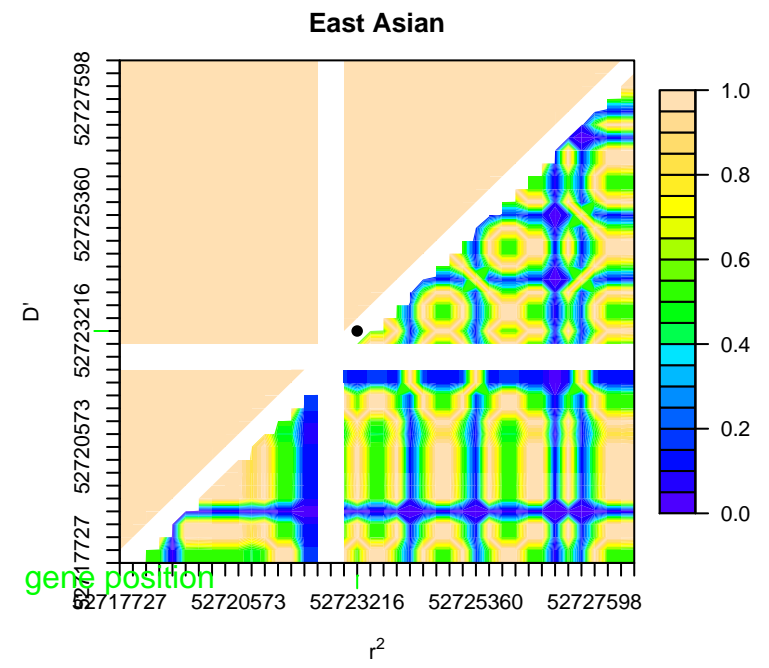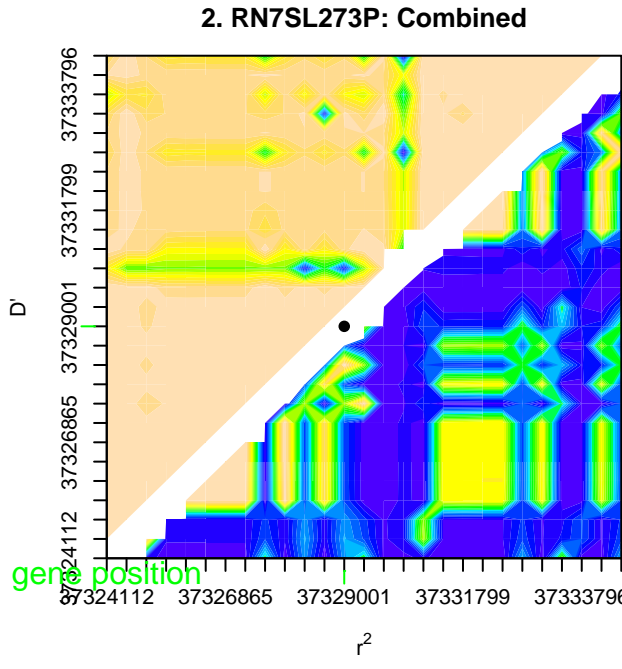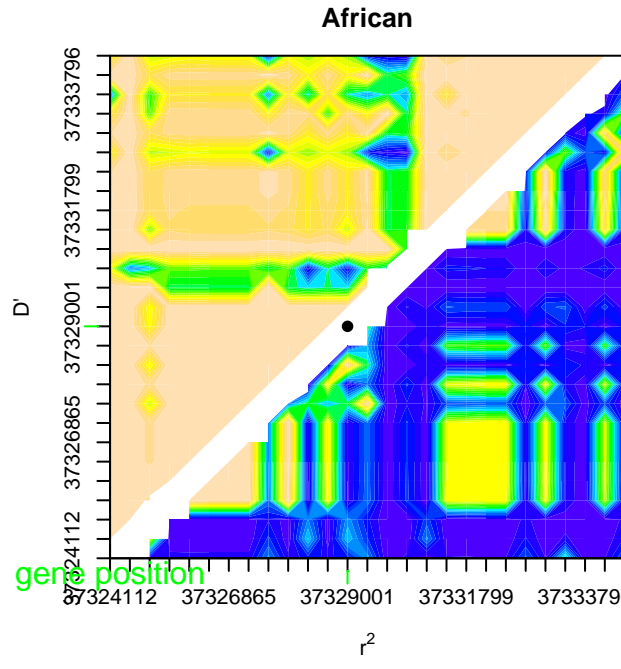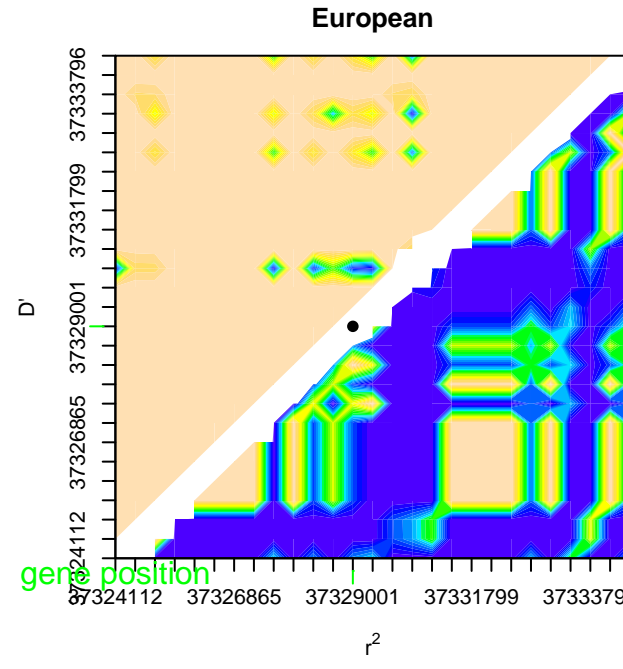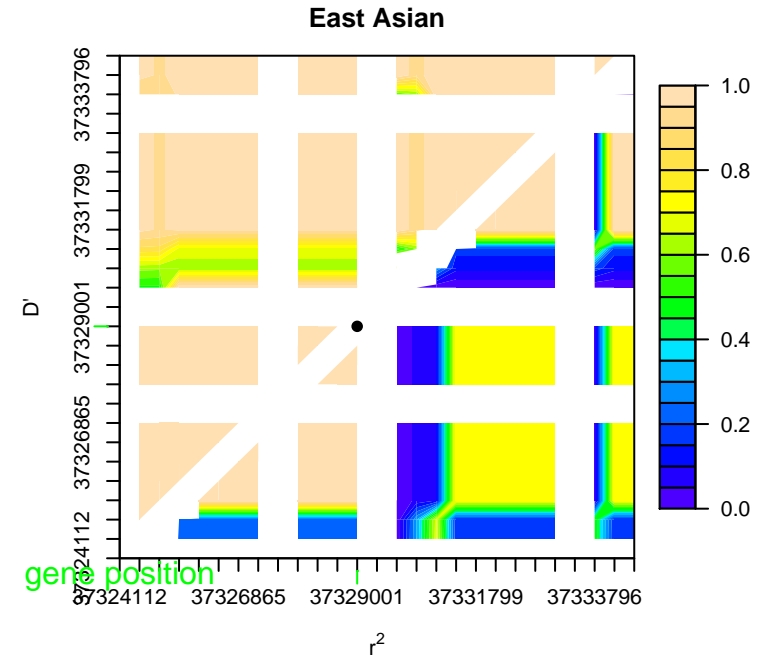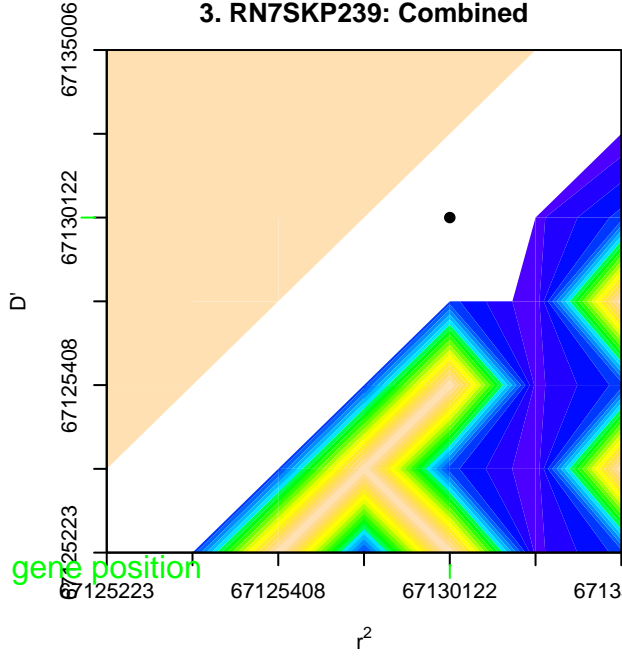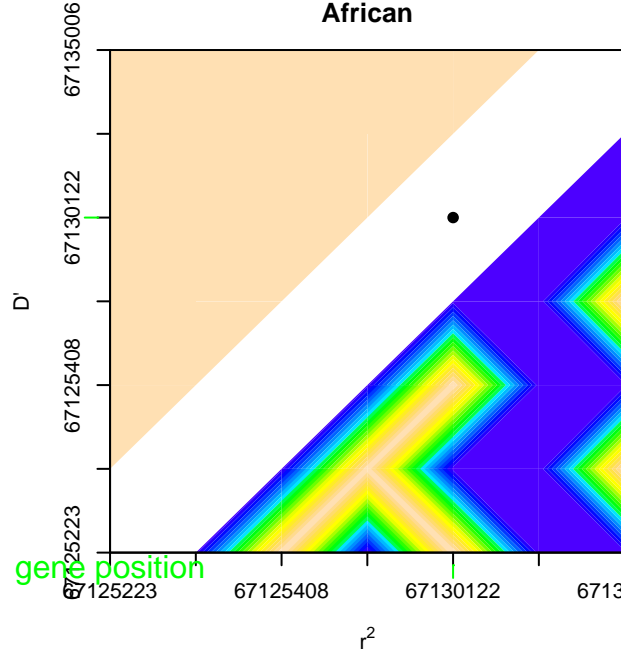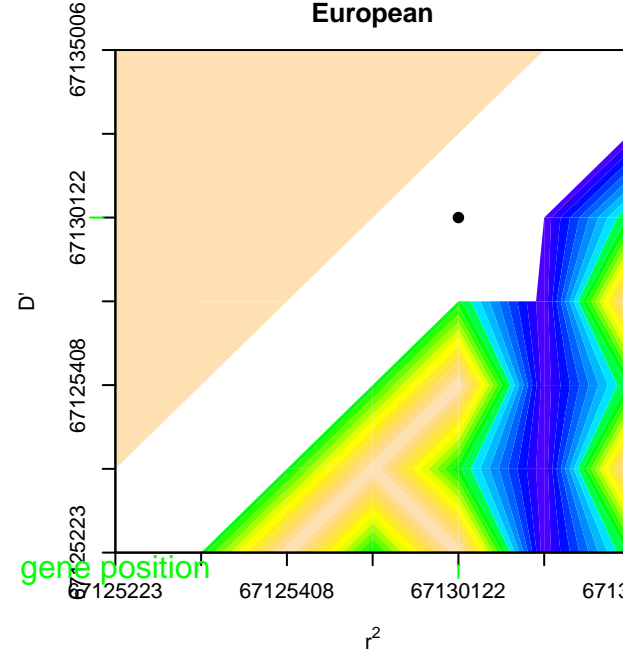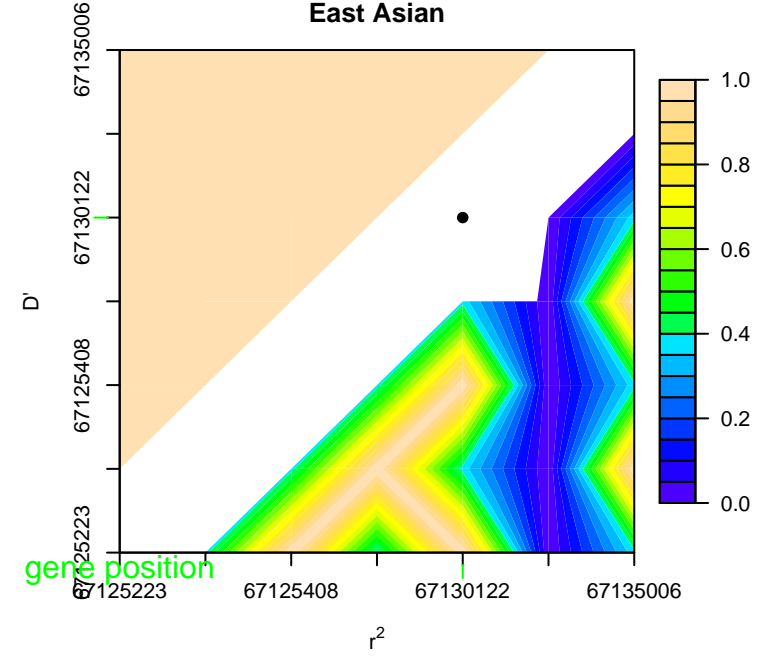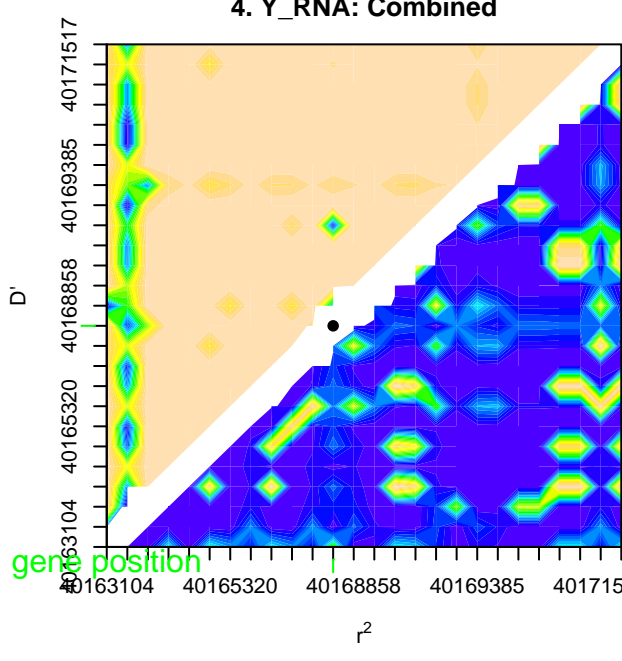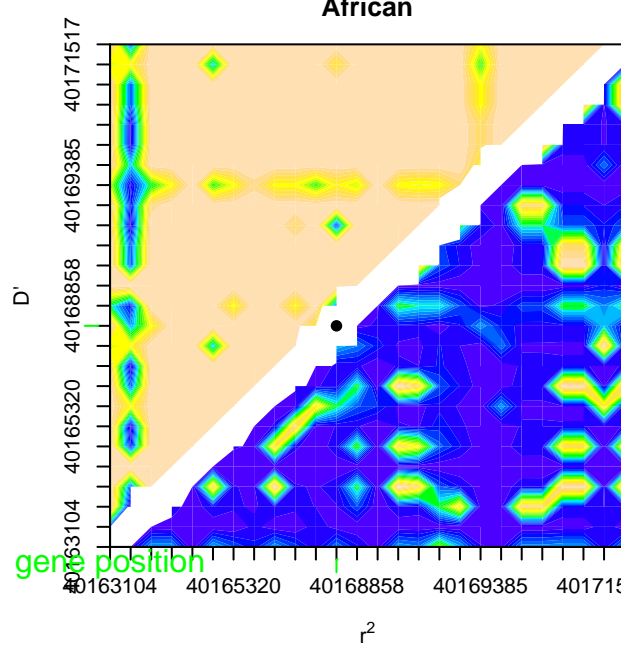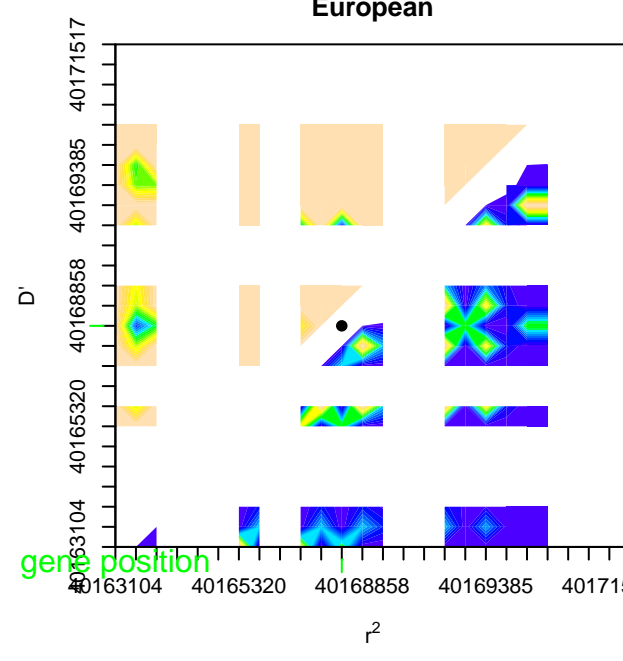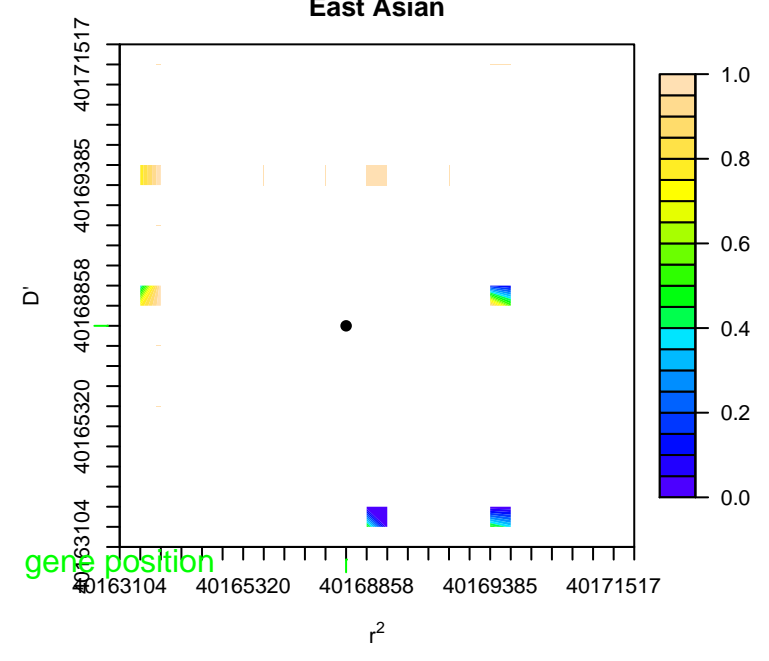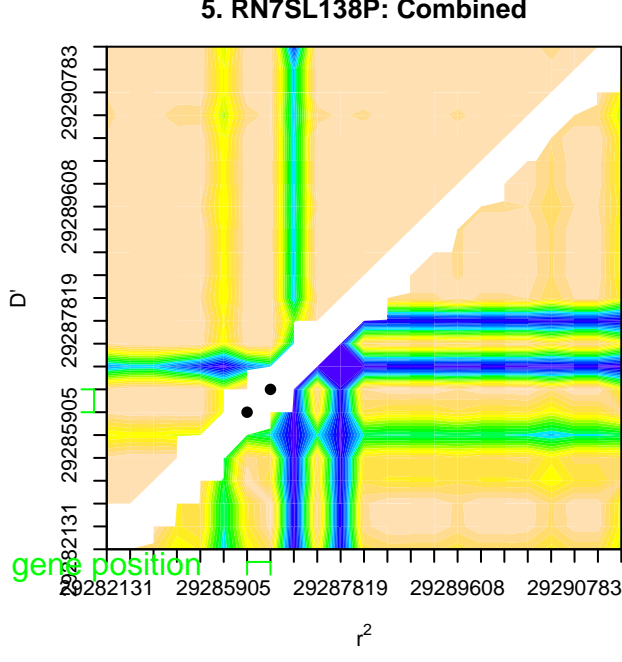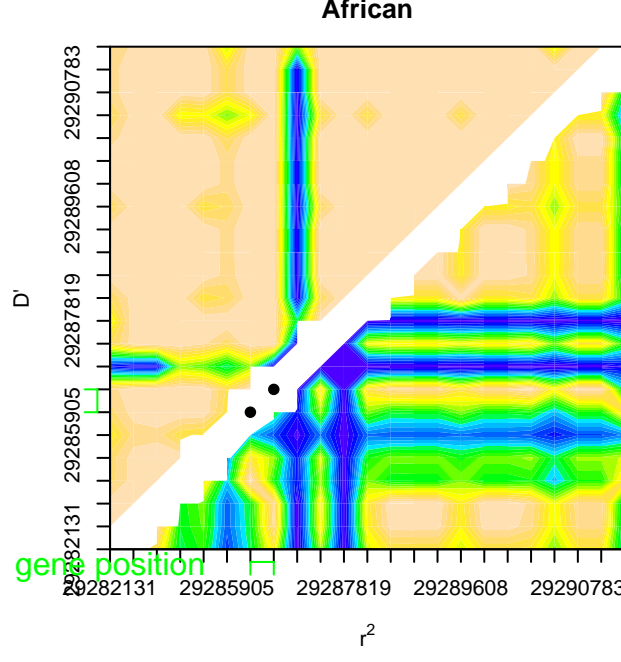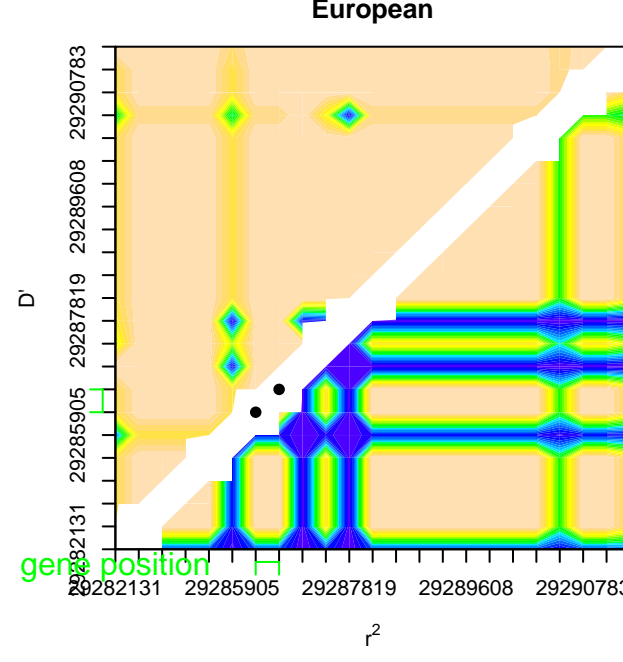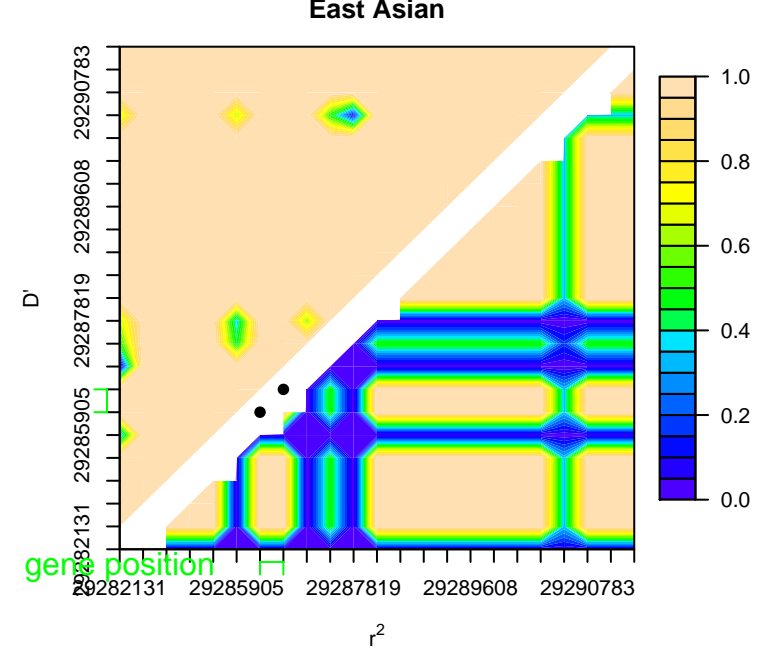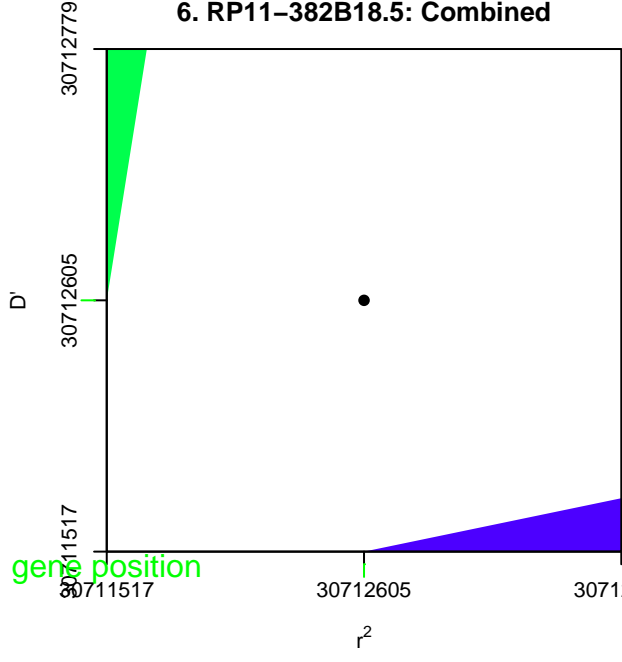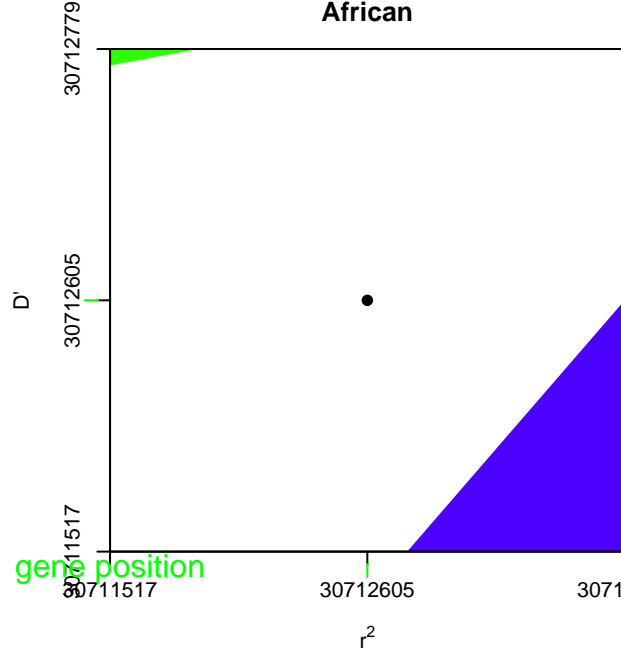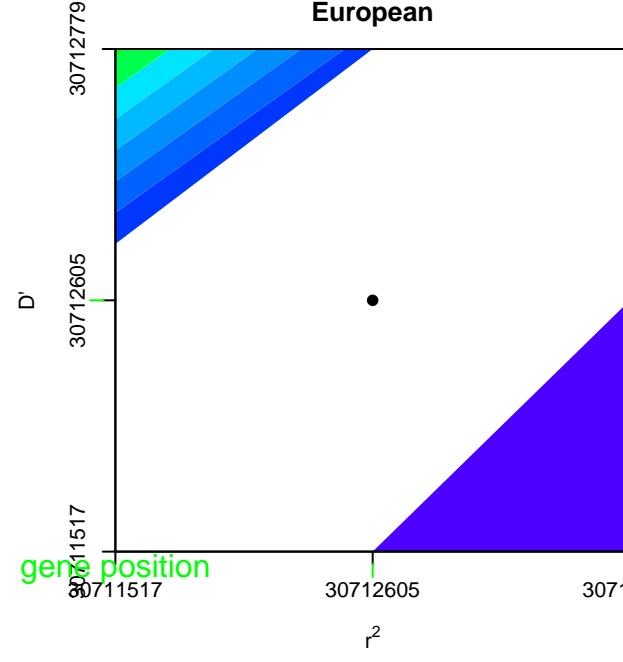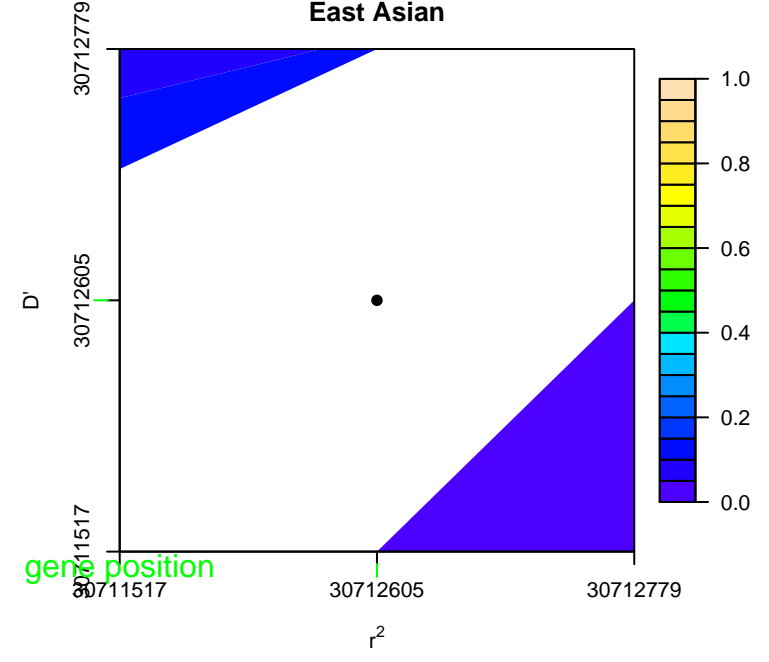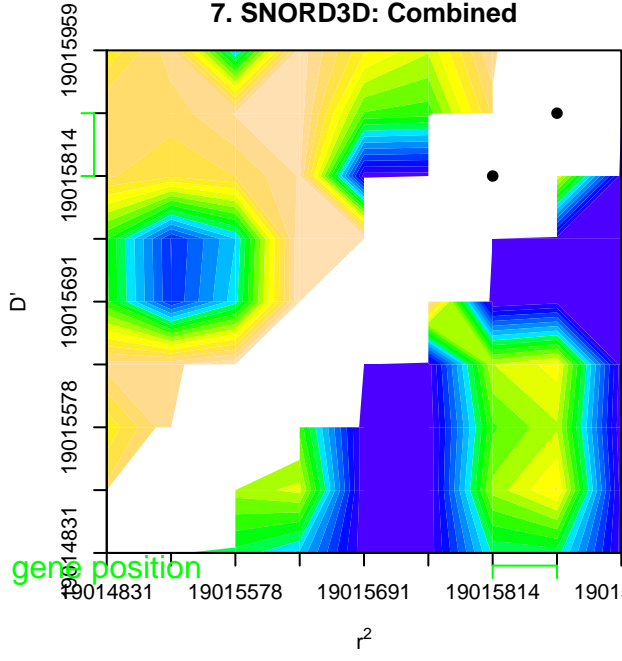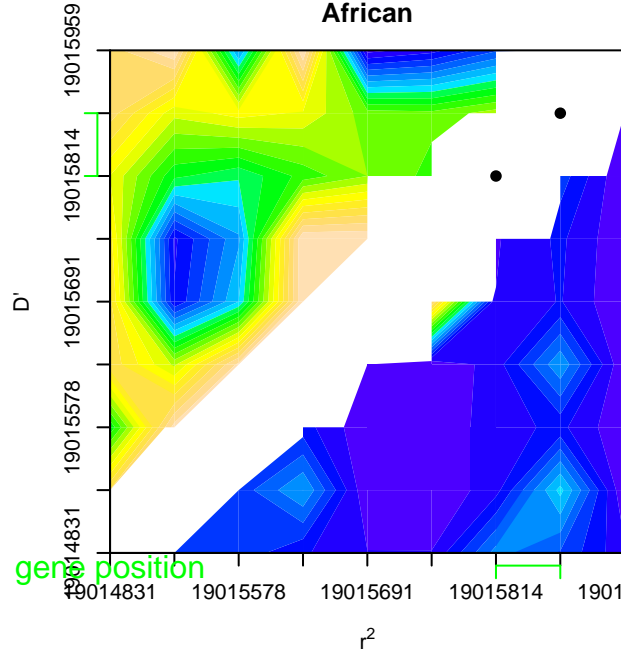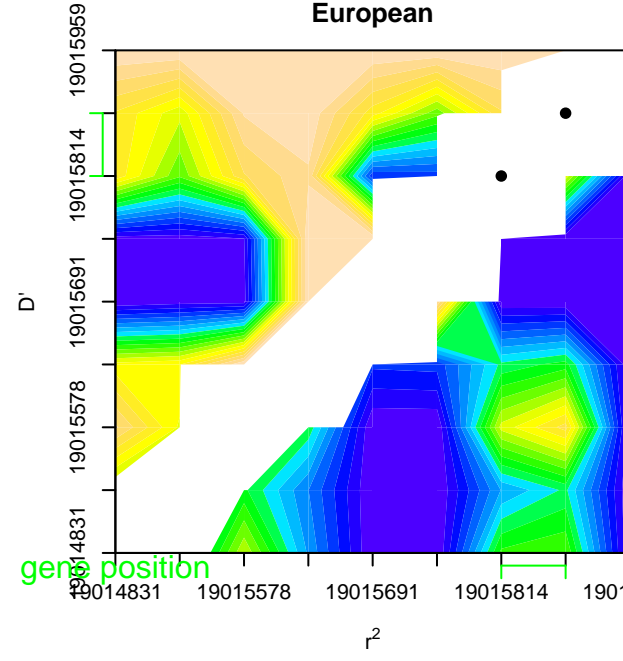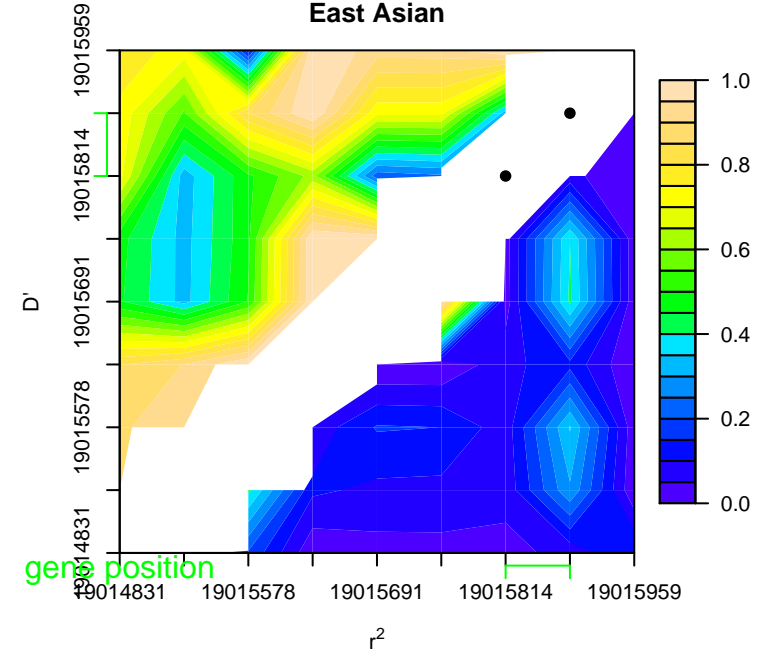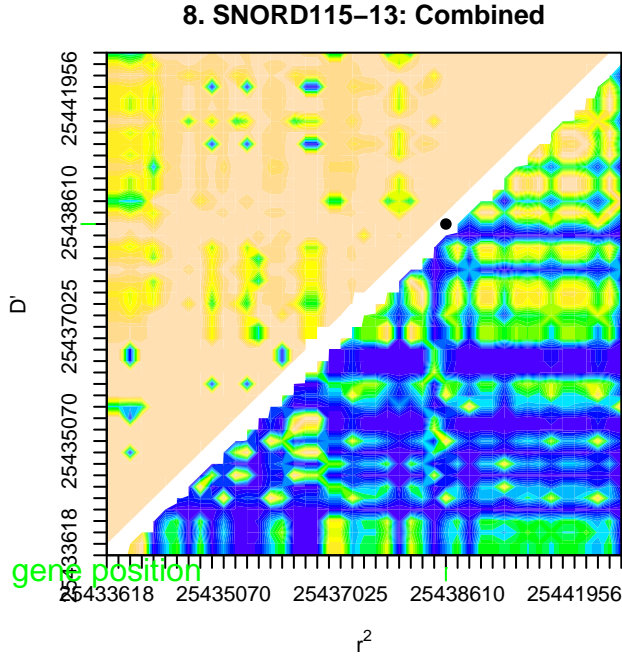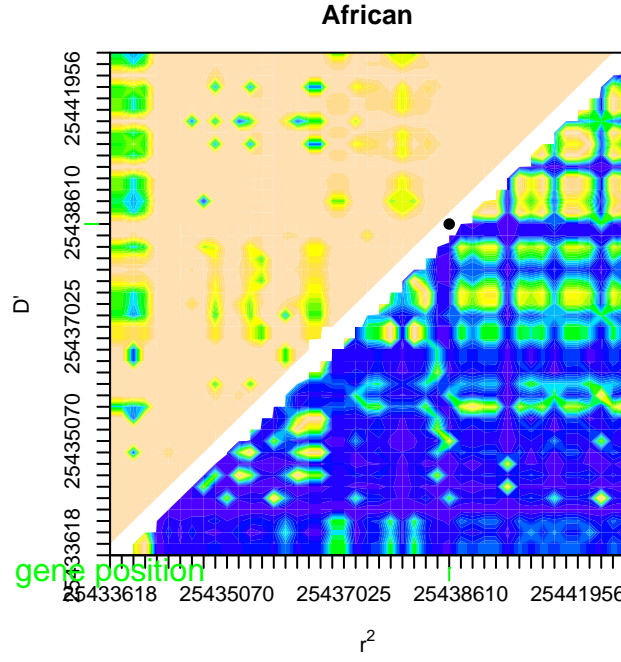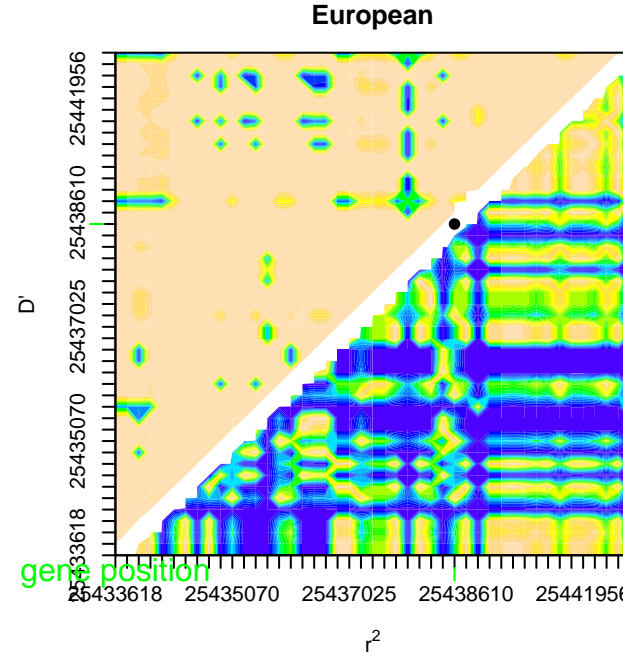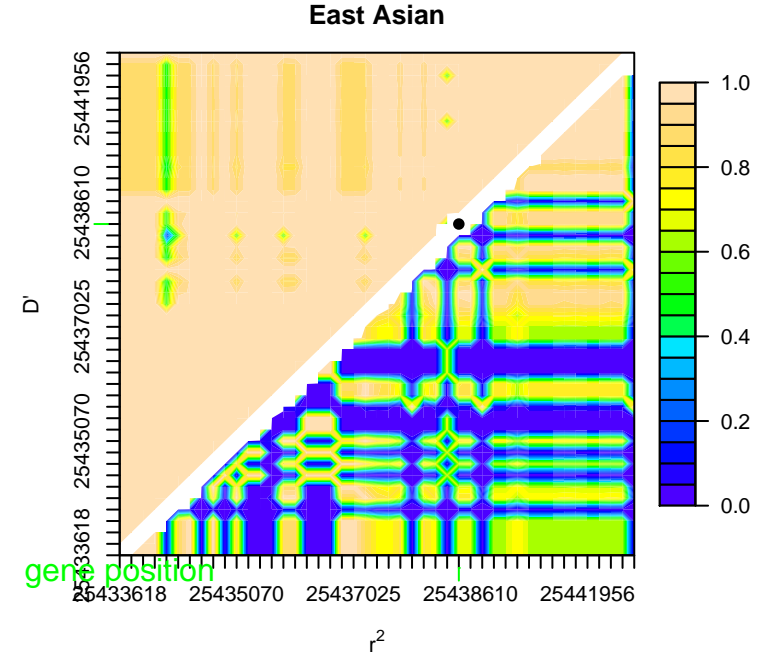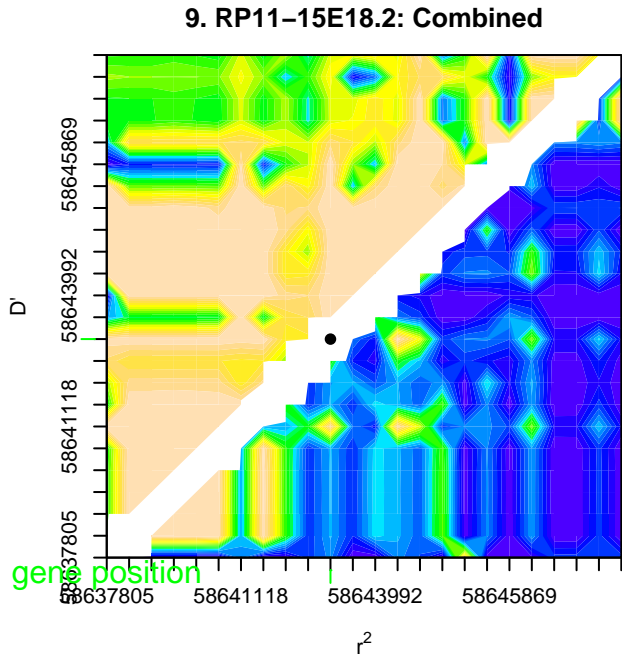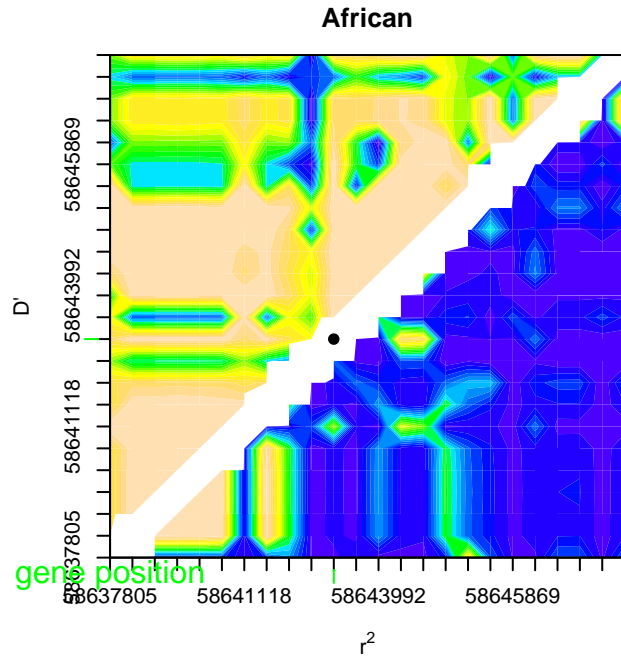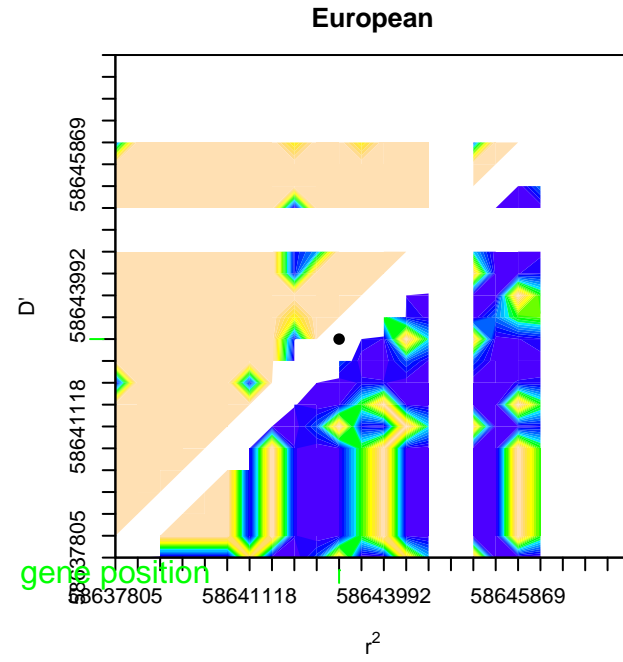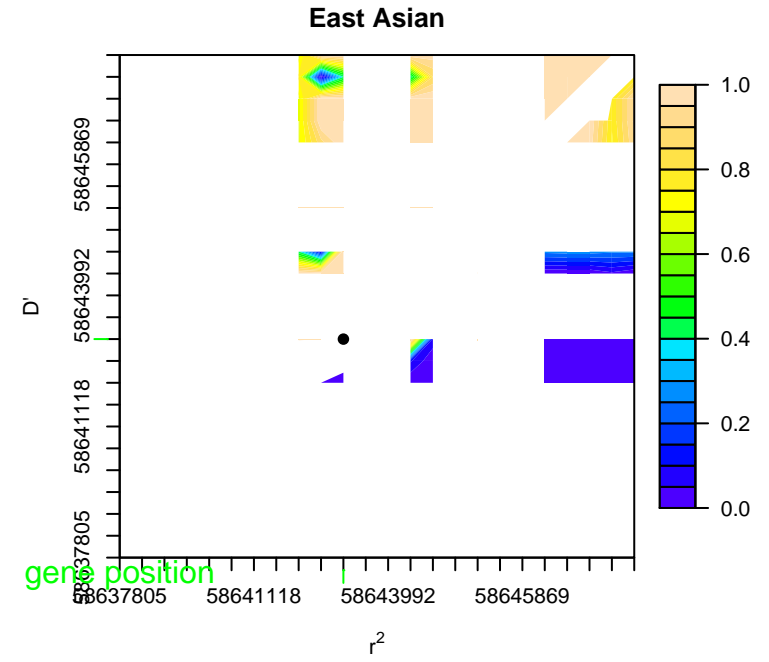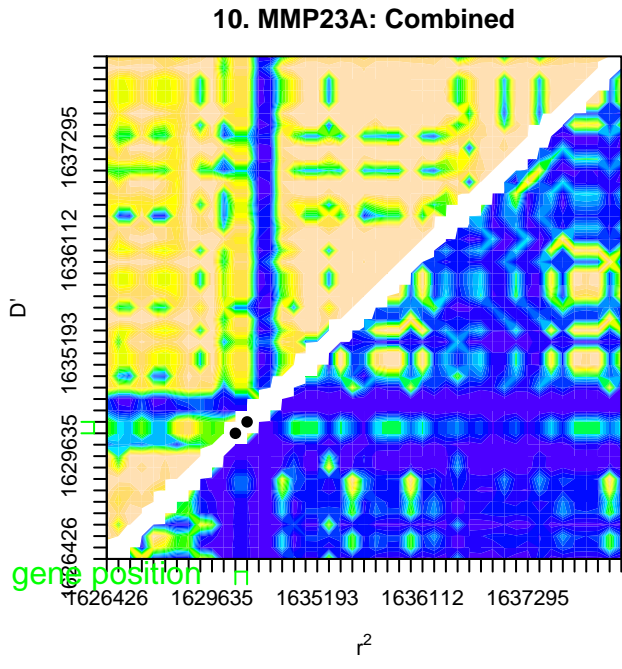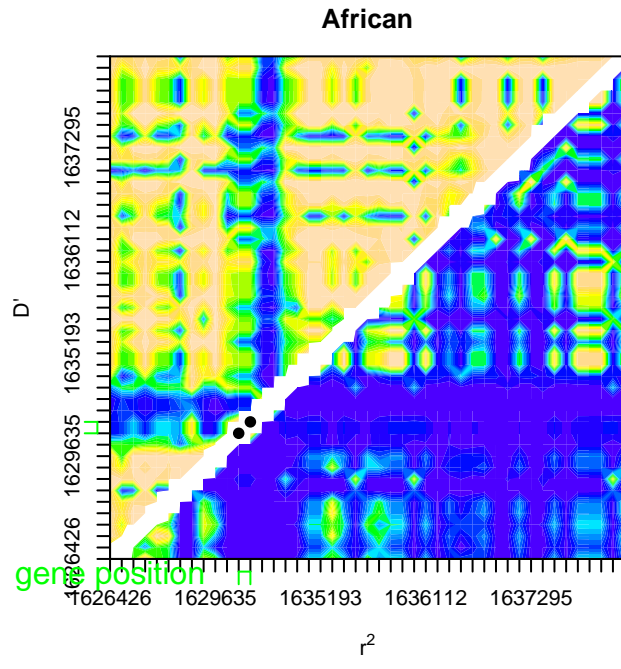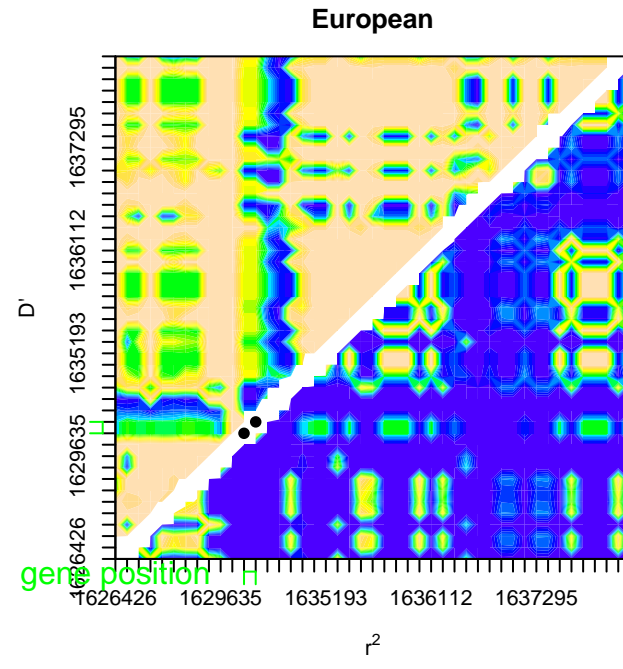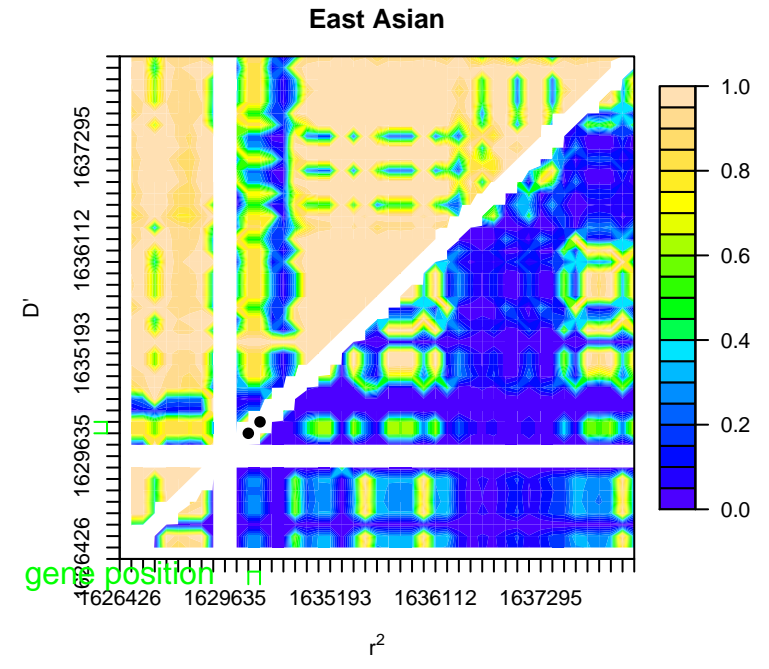

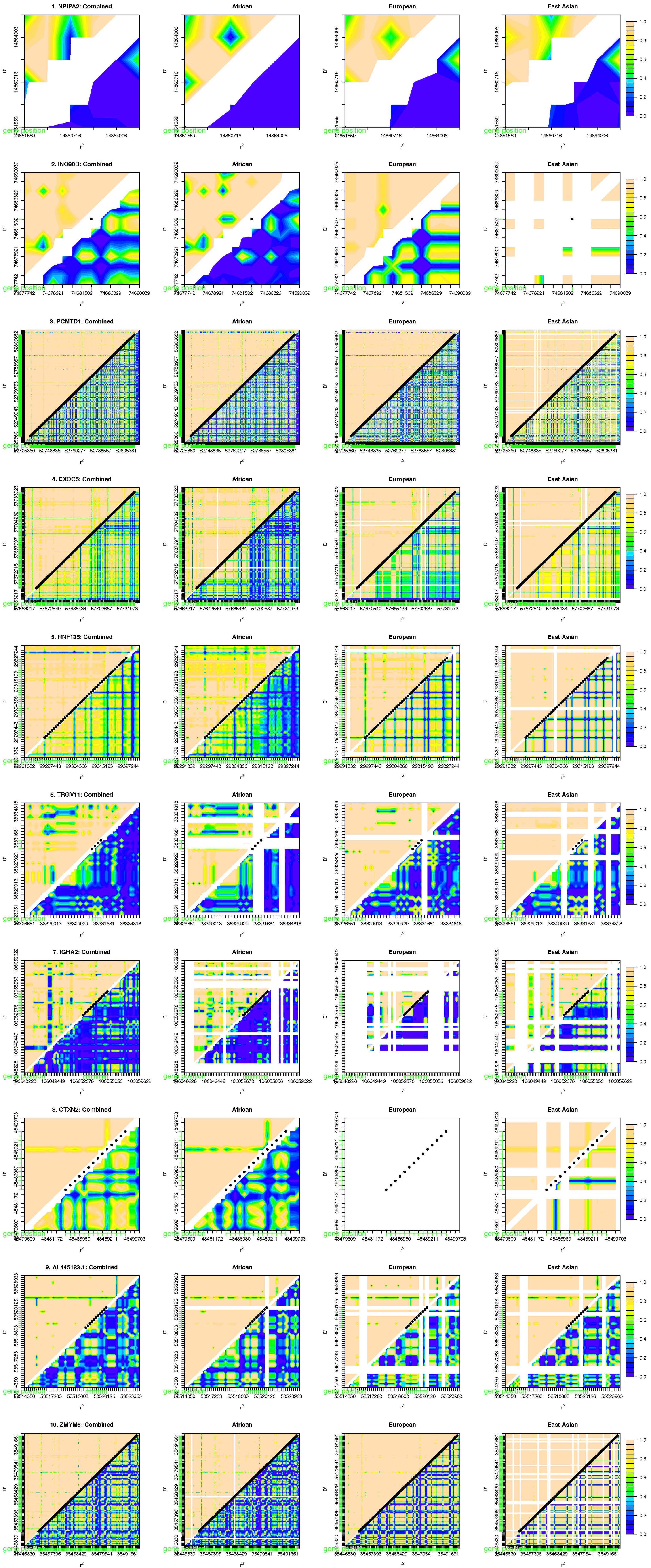

Supplement: S4 Fig — Linkage disequilibrium in the extended regions (±5,000) of the top FST estimates of coding and noncoding genes for the combined population: AFR, EUR, and EAS; (a) Noncoding genes; (b) Coding genes. (PDF) [file pone.0165870.s004.pdf]

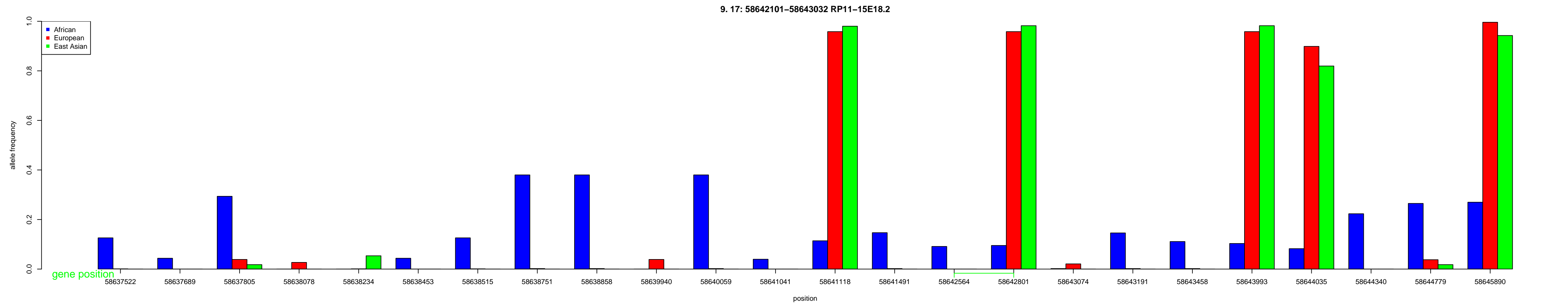

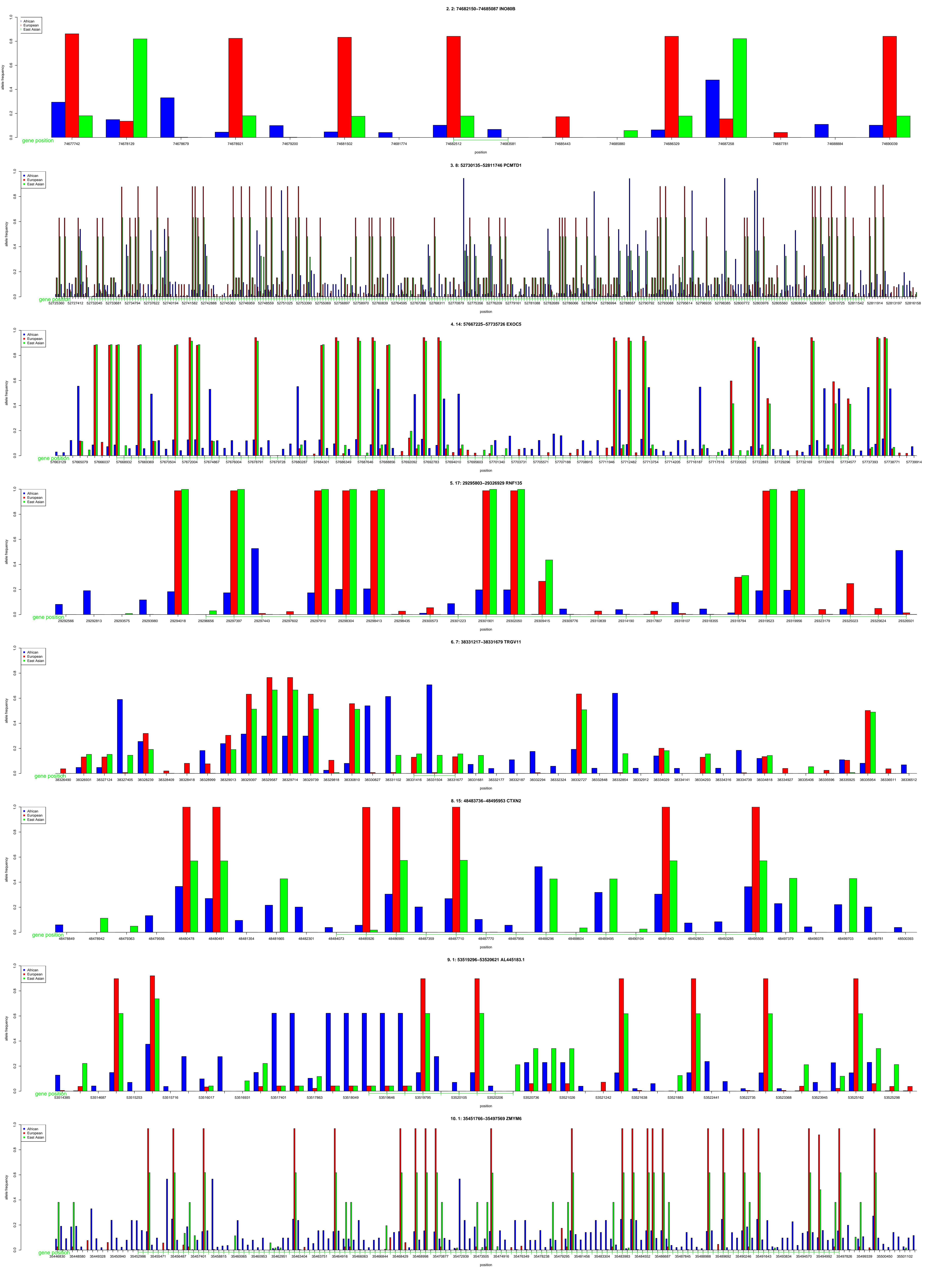

Supplement: S5 Fig — Derived allele frequency distribution of the extended regions (±5,000) of the top FST estimates of coding and noncoding genes; (a) Noncoding genes; (b) Coding genes. (PDF) [file pone.0165870.s005.pdf]

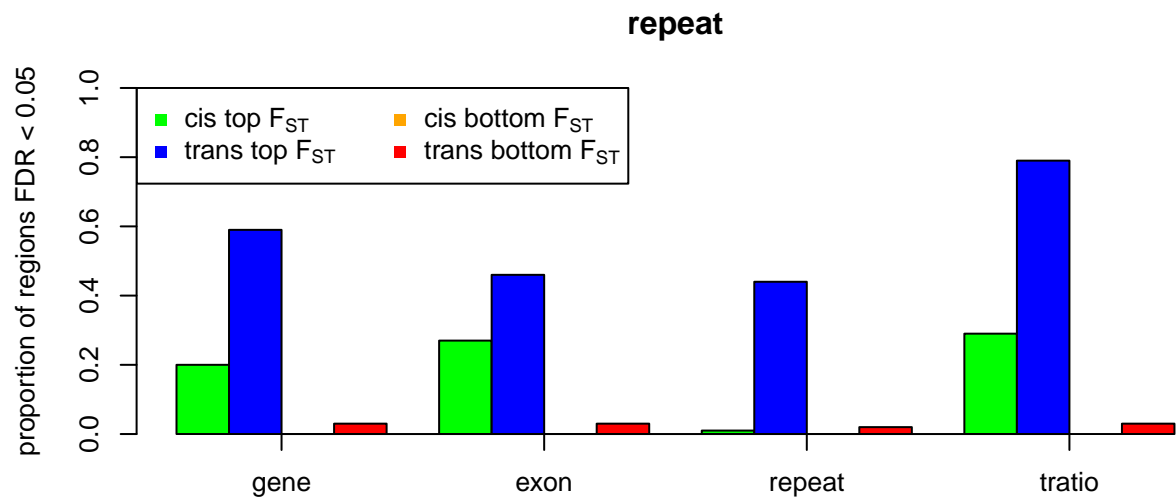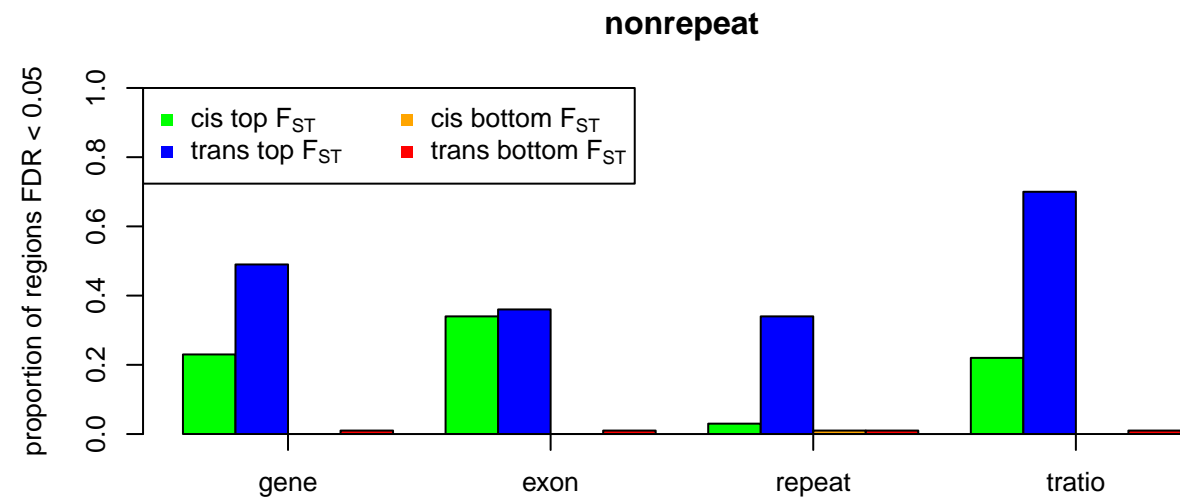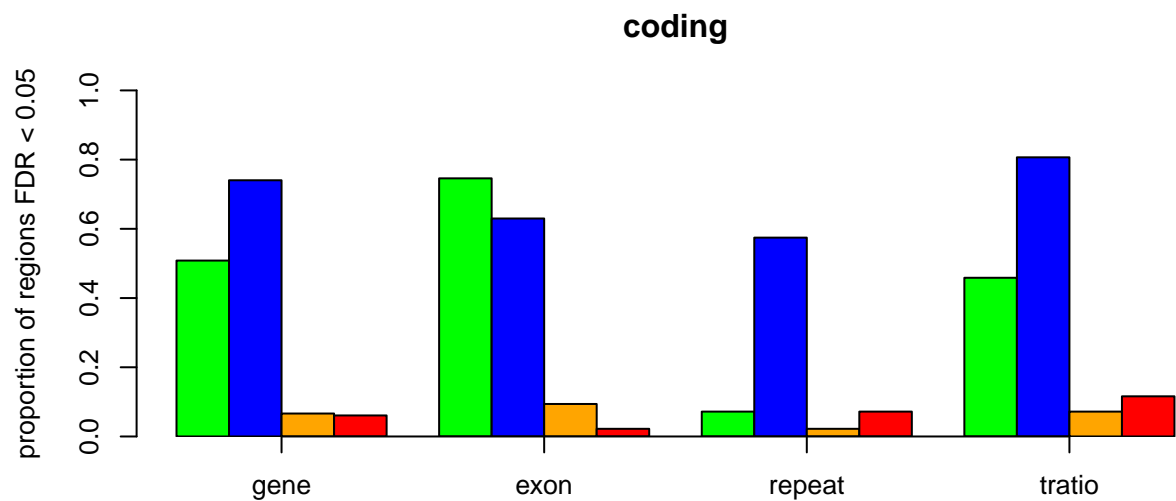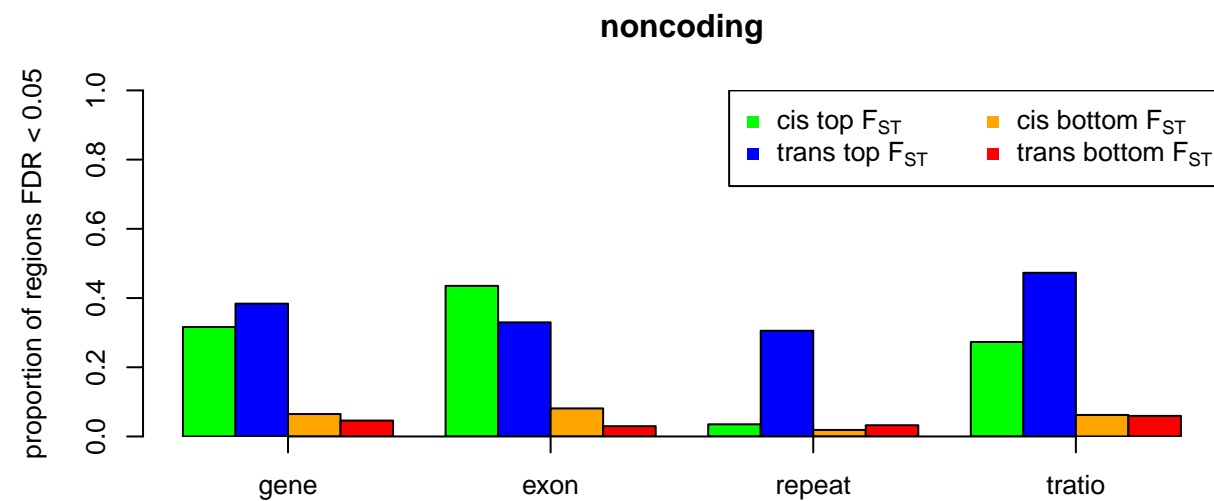

Supplement: S6 Fig — (PDF) [file pone.0165870.s006.pdf]

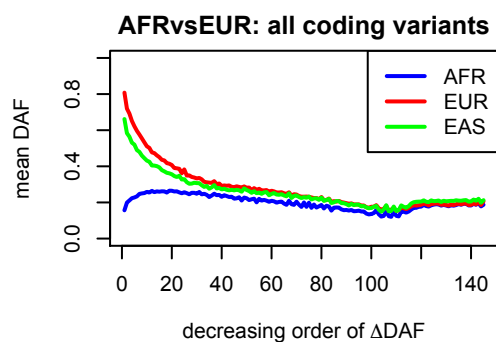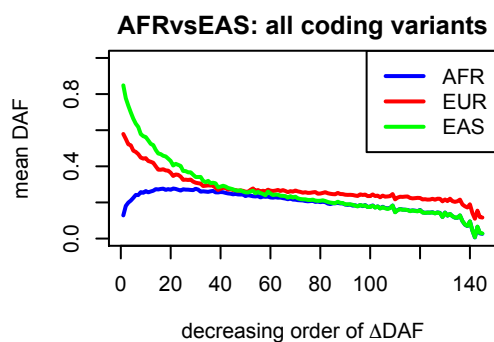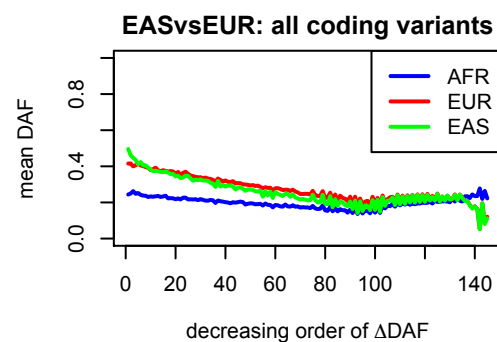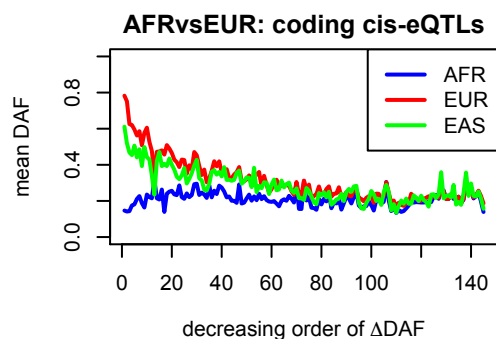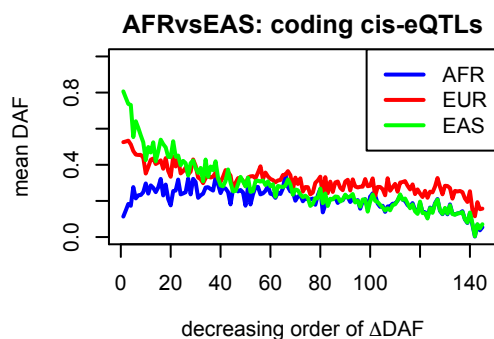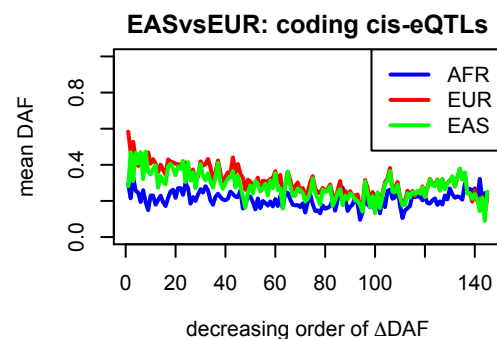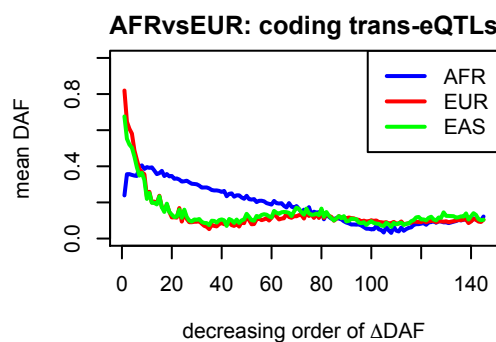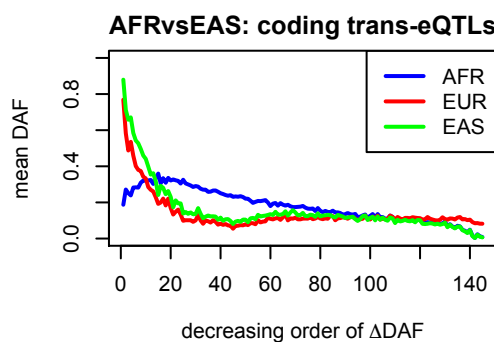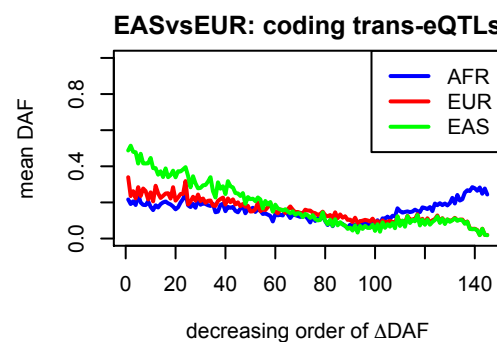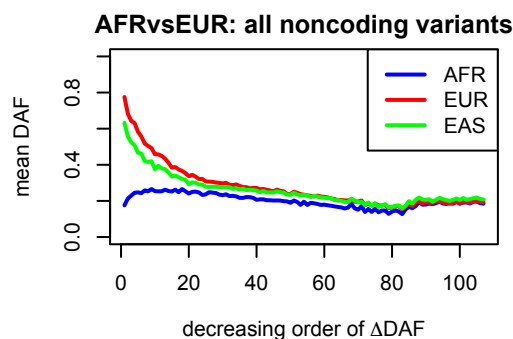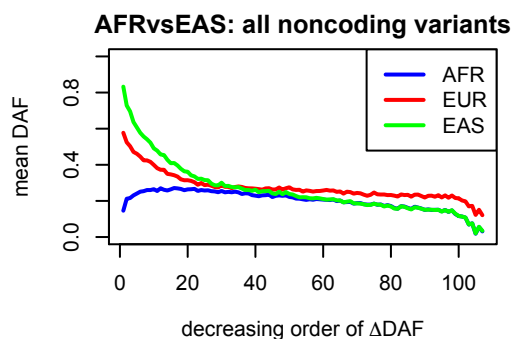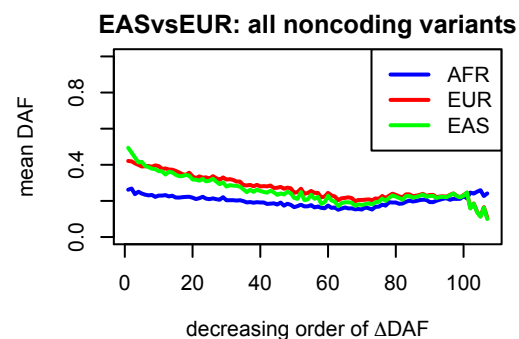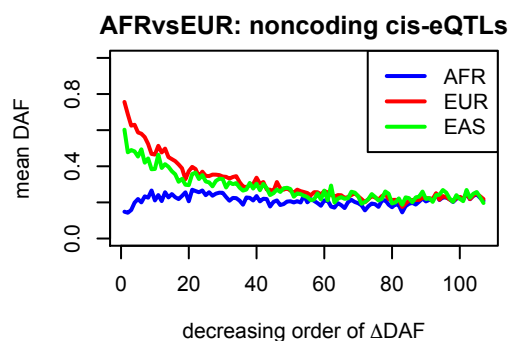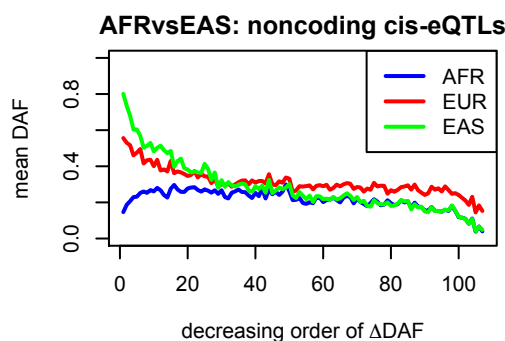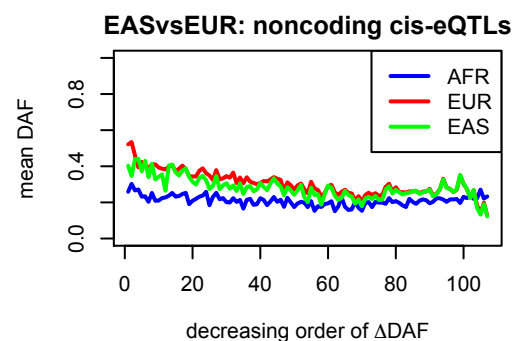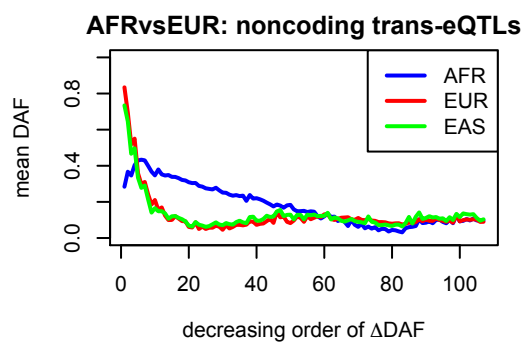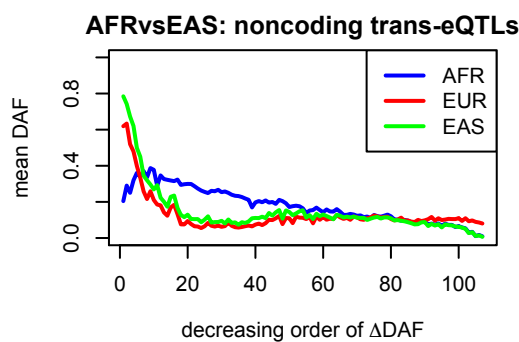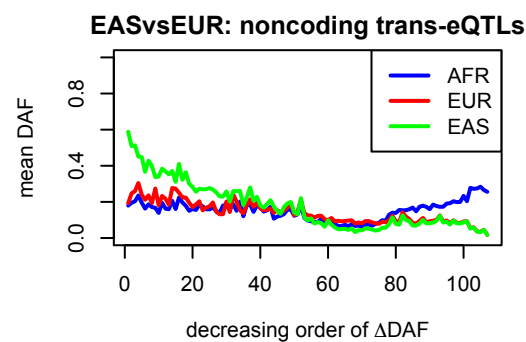

Supplement: S7 Fig — (PDF) [file pone.0165870.s007.pdf]
