# Supplementary material for: Evidence of Recent Intricate Adaptation in Human Populations
Source: PLoS One. 2016 Dec 19;11(12):e0165870. doi: 10.1371/journal.pone.0165870 (PMC5167553; doi:10.1371/journal.pone.0165870)
Supplement: S8 Fig — (PDF) [file pone.0165870.s008.pdf]

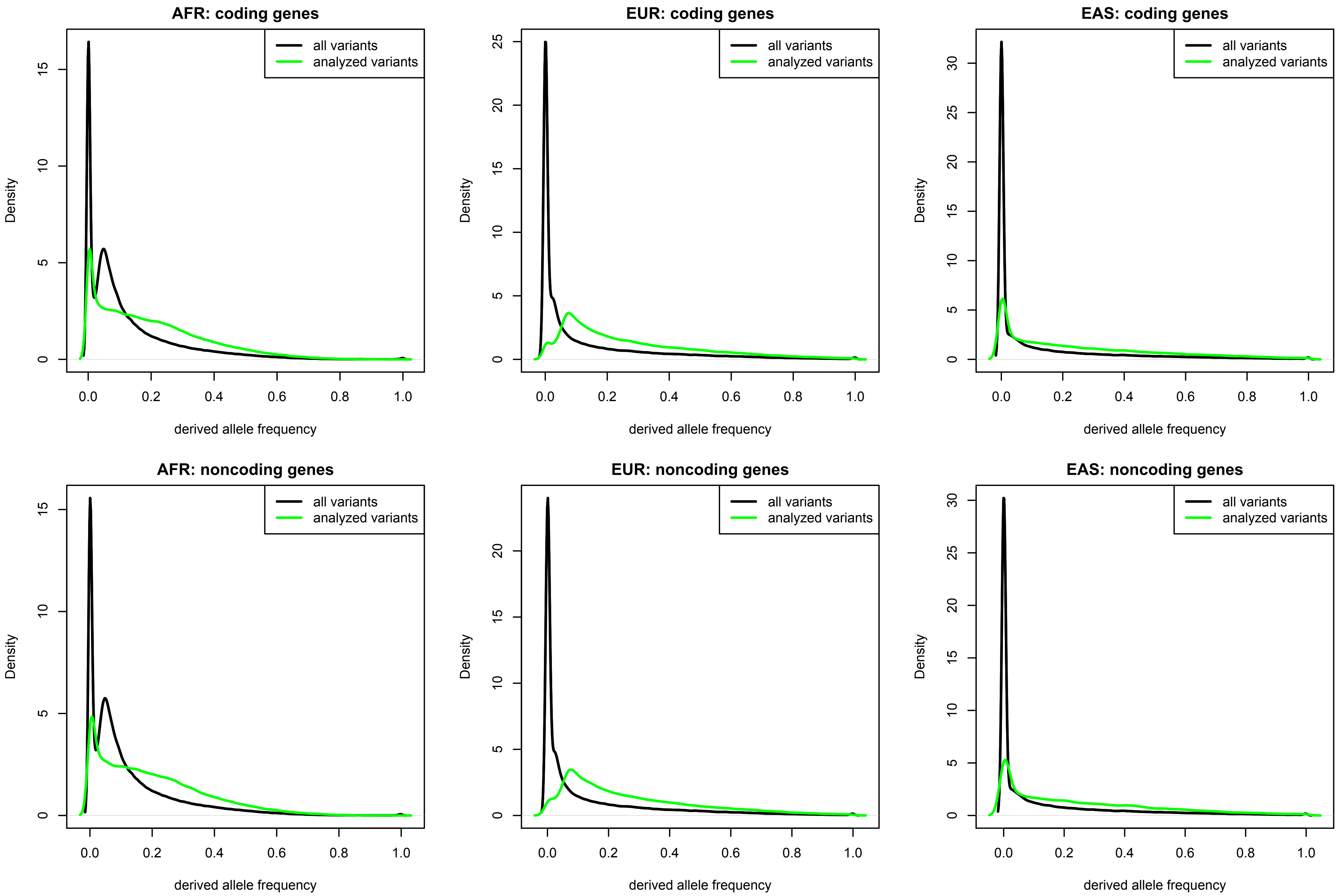

AFR: coding genes
EUR: coding genes
EAS: coding genes
AFR: noncoding genes
EUR: noncoding genes
EAS: noncoding genes
all variants
analyzed variants
Density
derived allele frequency
0.0
0.2
0.4
0.6
0.8
1.0
0
5
10
15
20
25
30
