# Supplementary material for: Evidence of Recent Intricate Adaptation in Human Populations
Source: PLoS One. 2016 Dec 19;11(12):e0165870. doi: 10.1371/journal.pone.0165870 (PMC5167553; doi:10.1371/journal.pone.0165870)
Supplement: S1 Table — (DOC) [file pone.0165870.s010.doc]

Supplementary Table

S1 Table. Summary of the number of eQTL that passed FDR <0.05 (tr: transcription ratio eQTL).

A. Population-specific variants in repeats (cis|trans).

|  | **rs#** | **repeats** | **chr** | **pos** | **gene** | **exon** | **repeat** | **tr** |
| --- | --- | --- | --- | --- | --- | --- | --- | --- |
| **1** | rs2413887 | Trf | chr15 | 48485926 | 0|32 | 0|60 | 1|239 | 0|302 |
| **2** | rs2675345 | L2b | chr15 | 48400199 | 0|35 | 0|47 | 0|183 | 0|294 |
| **3** | rs61658903 | AluY;dust;trf;AluJb | chr2 | 97538096 | 1|14 | 2|111 | 0|4 | 2|28 |
| **4** | rs8073072 | L2c | chr17 | 29350769 | 0|12 | 0|1 | 0|2 | 0|7 |
| **5** | rs12081911 | dust;AluSx1 | chr1 | 116938576 | 0|0 | 0|0 | 0|0 | 0|1 |
| **6** | rs6674304 | MIR | chr1 | 116887742 | 0|0 | 0|0 | 0|0 | 0|2 |
| **7** | rs12087680 | L2c | chr1 | 116950766 | 0|0 | 0|0 | 0|0 | 0|1 |
| **8** | rs73550618 | Trf | chr19 | 42418605 | 0|12 | 0|2 | 0|0 | 0|43 |
| **9** | rs2463171 | L1MD;dust | chr12 | 80106323 | 1|15 | 0|26 | 0|4 | 1|45 |
| **10** | rs191804091 | L1MC4 | chr17 | 18951783 | 2|27 | 18|80 | 0|4 | 1|97 |

B. Population-specific variants in non-repeat regions (cis|trans).

|  | **rs#** | **previous repeats** | **chr** | **pos** | **gene** | **exon** | **repeat** | **tr** |
| --- | --- | --- | --- | --- | --- | --- | --- | --- |
| **1** | rs2814778 | dust;trf | chr1 | 159174683 | 0|40 | 1|57 | 0|117 | 1|265 |
| **2** | rs1834640 | LTR91 | chr15 | 48392165 | 0|40 | 0|59 | 0|190 | 0|201 |
| **3** | rs7749696 | AluSg7;dust;trf | chr6 | 10654425 | 0|2 | 3|4 | 0|0 | 2|9 |
| **4** | rs2470102 | AluSg;trf;dust | chr15 | 48433494 | 0|47 | 0|122 | 0|136 | 0|308 |
| **5** | rs150515964 | dust;trf | chr6 | 104886714 | 0|1 | 0|0 | 0|0 | 0|0 |
| **6** | rs7753890 | dust;trf | chr6 | 136516257 | 0|0 | 1|5 | 0|6 | 1|13 |
| **7** | rs6875659 | MIR;MamSINE1 | chr5 | 175158653 | 0|5 | 0|2 | 0|0 | 0|15 |
| **8** | rs4790359 | dust | chr17 | 2631985 | 0|5 | 0|3 | 0|2 | 0|55 |
| **9** | rs7211872 | Kanga2_a | chr17 | 58550725 | 0|3 | 0|25 | 0|3 | 0|24 |
| **10** | rs6570067 | L1ME4a | chr6 | 136496831 | 0|10 | 0|11 | 0|8 | 2|31 |

C. Variants of minor allele frequency higher than 0.05 in noncoding genes (cis|trans).

|  | **gene name** | **chr** | **gene** | **exon** | **repeat** | **tr** |
| --- | --- | --- | --- | --- | --- | --- |
| **1** | RP11-11C20.3 | chr8 | 2|2 | 7|0 | 0|0 | 3|1 |
| **2** | RN7SL273P | chr6 | 0|13 | 0|4 | 0|0 | 1|20 |
| **3** | RN7SKP239 | chr11 | 0|1 | 1|0 | 0|0 | 0|0 |
| **4** | Y_RNA | chr7 | 0|5 | 0|9 | 0|3 | 0|73 |
| **5** | RN7SL138P | chr17 | 7|55 | 13|109 | 0|5 | 4|111 |
| **6** | RP11-382B18.5 | chr15 | 2|1 | 1|2 | 0|0 | 2|0 |
| **7** | SNORD3D | chr17 | 3|1 | 3|0 | 0|0 | 4|5 |
| **8** | SNORD115-13 | chr15 | 0|0 | 0|0 | 0|0 | 0|2 |
| **9** | RP11-15E18.2 | chr17 | 0|0 | 0|0 | 0|0 | 0|9 |
| **10** | MMP23A | chr1 | 0|0 | 6|0 | 0|0 | 0|2 |

D. Variants of minor allele frequency higher than 0.05 in coding genes (cis|trans).

|  | **gene name** | **chr** | **gene** | **exon** | **repeat** | **tr** |
| --- | --- | --- | --- | --- | --- | --- |
| **1** | NPIPA2 | chr16 | 0|0 | 17|1 | 0|0 | 0|0 |
| **2** | INO80B | chr2 | 2|0 | 5|0 | 0|0 | 2|0 |
| **3** | PCMTD1 | chr8 | 404|301 | 1442|0 | 0|52 | 787|232 |
| **4** | EXOC5 | chr14 | 0|1 | 632|0 | 0|0 | 15|201 |
| **5** | RNF135 | chr17 | 97|812 | 236|1691 | 0|73 | 8|1249 |
| **6** | TRGV11 | chr7 | 0|4 | 6|8 | 0|10 | 0|3 |
| **7** | IGHA2 | chr14 | 0|31 | 0|21 | 0|1 | 0|23 |
| **8** | CTXN2 | chr15 | 0|162 | 0|1017 | 1|627 | 0|535 |
| **9** | AL445183.1 | chr1 | 0|3 | 5|16 | 0|1 | 0|49 |
| **10** | ZMYM6 | chr1 | 4|409 | 89|444 | 0|33 | 27|1186 |
